# Structure Analysis Uncovers a Highly Diverse but Structurally Conserved Effector Family in Phytopathogenic Fungi

**DOI:** 10.1371/journal.ppat.1005228

**Published:** 2015-10-27

**Authors:** Karine de Guillen, Diana Ortiz-Vallejo, Jérome Gracy, Elisabeth Fournier, Thomas Kroj, André Padilla

**Affiliations:** 1 INSERM U1054, Centre de Biochimie Structurale, Montpellier, France; 2 CNRS UMR5048, Montpellier University, Montpellier, France; 3 INRA, BGPI, Biology and Genetics of Plant-Pathogen Interactions, Campus International de Baillarguet, Montpellier, France; 4 CIRAD, BGPI, Biology and Genetics of Plant-Pathogen Interactions, Campus International de Baillarguet, Montpellier, France; Purdue University, UNITED STATES

## Abstract

Phytopathogenic ascomycete fungi possess huge effector repertoires that are dominated by hundreds of sequence-unrelated small secreted proteins. The molecular function of these effectors and the evolutionary mechanisms that generate this tremendous number of singleton genes are largely unknown. To get a deeper understanding of fungal effectors, we determined by NMR spectroscopy the 3-dimensional structures of the *Magnaporthe oryzae* effectors AVR1-CO39 and AVR-Pia. Despite a lack of sequence similarity, both proteins have very similar 6 β-sandwich structures that are stabilized in both cases by a disulfide bridge between 2 conserved cysteins located in similar positions of the proteins. Structural similarity searches revealed that AvrPiz-t, another effector from *M*. *oryzae*, and ToxB, an effector of the wheat tan spot pathogen *Pyrenophora tritici-repentis* have the same structures suggesting the existence of a family of sequence-unrelated but structurally conserved fungal effectors that we named MAX-effectors (***M*** *agnaporthe* **A**vrs and To**x**B like). Structure-informed pattern searches strengthened this hypothesis by identifying MAX-effector candidates in a broad range of ascomycete phytopathogens. Strong expansion of the MAX-effector family was detected in *M*. *oryzae* and *M*. *grisea* where they seem to be particularly important since they account for 5–10% of the effector repertoire and 50% of the cloned avirulence effectors. Expression analysis indicated that the majority of *M*. *oryzae* MAX-effectors are expressed specifically during early infection suggesting important functions during biotrophic host colonization. We hypothesize that the scenario observed for MAX-effectors can serve as a paradigm for ascomycete effector diversity and that the enormous number of sequence-unrelated ascomycete effectors may in fact belong to a restricted set of structurally conserved effector families.

## Author Summary

Fungal plant pathogens are of outstanding economic and ecological importance and cause destructive diseases on many cultivated and wild plants. Effector proteins that are secreted during infection to manipulate the host and to promote disease are a key element in fungal virulence. Phytopathogenic fungi possess huge effector repertoires that are dominated by hundreds of sequence-unrelated small secreted proteins. The molecular functions of this most important class of fungal effectors and the evolutionary mechanisms that generate this tremendous numbers of apparently unrelated proteins are largely unknown. By investigating the 3-dimensional structures of effectors from the rice blast fungus *M*. *oryzae*, we discovered an effector family comprising structurally conserved but sequence-unrelated effectors from *M*. *oryzae* and the phylogenetically distant wheat pathogen *Pyrenophora tritici-repentis* that we named MAX-effectors (*M*. *oryzae* Avrs and ToxB). Structure-informed searches of whole genome sequence databases suggest that MAX-effectors are present at low frequencies and with a patchy phylogenetic distribution in many ascomycete phytopathogens. They underwent strong lineage-specific expansion in fungi of the *Pyriculariae* family that contains *M*. *oryzae* where they seem particularly important during biotrophic plant colonization and account for 50% of the cloned Avr effectors and 5–10% of the effector repertoire. Based on our results on the MAX-effectors and the widely accepted concept that fungal effectors evolve according to a birth-and-death model we propose the hypothesis that the majority of the immense numbers of different ascomycete effectors could in fact belong to a limited set of structurally defined families whose members are phylogenetically related.

## Introduction

Pathogenic microorganisms have to cope with the immune system of their host and therefore deploy measures to hide their presence, disturb host immunity or inactivate defense responses. In all these strategies, proteins secreted by the pathogen during infection and acting on host proteins and cellular processes play a key role [1–3]. These proteinaceous virulence factors named effectors act either extra-cellularly or inside host cells and can possess, depending on the microorganism, very different molecular features.

In fungal pathogens, the main class of effectors are small secreted proteins of less than 200 amino acids expressed specifically during infection and often rich in cysteins [4–6]. Genome sequencing and expression analysis identified hundreds of such effector candidates in individual plant pathogenic fungal species. Few of them, mainly those acting extra-cellularly, are widely distributed among phytopathogenic fungi and contain known motifs or domains, such as NLPs (necrosis and ethylene-inducing peptide 1 (Nep1)-like proteins), LysM domain-containing proteins or protease inhibitors [5,6]. The vast majority of the fungal effectors do not share sequence similarities with other proteins and do not contain conserved motifs. This is very different from the situation in other phytopathogens and in particular oomyctes, an important class of plant pathogens that have similar lifestyles and infection strategies and whose virulence relies also on large effector repertoires. In oomycete pathogens, large families of cytoplasmic effectors with hundreds of members in individual species are defined by the presence of the RXLR or the LFLAK host cell translocation motifs [7–9]. The effector domains of these RXLR and Crinkler (CRN) effectors that mediate virulence functions are highly diversified but contain, in the majority of cases, conserved motifs or domains that are shared between effectors from the same or other species allowing their classification in distinct families. On the contrary, most fungal effectors are species-specific while few are lineage specific and occur in closely related species. In most phytopathogenic fungi, no large effector gene families were identified [5,6]. The majority of their effectors are singletons and a small proportion belongs to small paralogous groups of rarely more than 3 members. Effector repertoires dominated by gene families of large size counting more than 5 members were only detected in particular cases such as powdery mildew and rust fungi lineages [10–13]. Due to their high diversity and the lack of similarity with other proteins, the mode of action and the role in infection of fungal effectors have to be elucidated case by case and remain still largely unknown [5,6]. In addition, this tremendous diversity raises the question of the evolutionary trajectories of fungal effectors that do not show traces of common origins.

Rice blast disease caused by the ascomycete fungus *M*. *oryzae* is present in all rice growing areas and causes important harvest losses. Since rice is the main source of calories for half of the human population and since disease control strategies are frequently overcome by the pathogen due to its high genetic plasticity, blast is considered one of the most dangerous plant diseases threatening global food security and hampering attempts to increase rice yield in many parts of the world [14–16]. Due to its economic importance, the status of the host plant rice as a model plant and the ease of cultivation and genetic manipulation of *M*. *oryzae*, blast disease has become a model for the molecular and genetic investigation of fungal plant diseases [14]. In particular, molecular mechanisms of fungal disease development were studied intensively in *M*. *oryzae* uncovering important features of fungal virulence [17,18]. Key steps in infection by *M*. *oryzae* are (i) penetration into epidermal cells by the breakage of the leaf cuticle and epidermal cell walls by an appressorium, a specialized unicellular structure, (ii) biotrophic growth inside the first invaded host cells, followed by (iii) necrotrophic growth associated with active killing of host tissue and the development of disease symptoms and finally, (iv) clonal reproduction and sporulation.

Effectors and in particular cytoplasmic effectors are key elements in *M*. *oryzae* virulence and particularly important during the biotrophic phase of infection [6,19,20]. However, the function of individual effectors in the infection process has only been established for the LysM effector SLP1 that sequesters chitin fragments and thereby interferes with their recognition by the rice chitin receptor CEBiP, and AvrPiz-t that interferes with host immunity by inhibiting the E3 ubiquitin ligase APIP6 [21,22]. Mutant analysis aiming to demonstrate that individual effectors are important for virulence have often been unsuccessful, probably due to functional redundancy among effectors [23,24]. Approximately 700 of the 1300–1500 secreted proteins encoded in the *M*. *oryzae* genome are considered effector candidates according to their size of less than 200 amino acids and their lack of homology to proteins of known function [25,26]. Hundreds of them were found to be expressed during appressoria formation or infection [23,26–28].

Some effectors are recognized in certain plant accessions by immune receptors localized either at the plasma membrane or in the cytosol leading to the induction of strong defense responses and resistance to pathogen isolates possessing this effector [29]. The recognized effector is, in these cases, named an avirulence (Avr) protein. In *M*. *oryzae*, 8 different effectors acting as Avr proteins named PWL2, AVR-Pia, AVR1-CO39, AVR-Pii, AVR-Pik, AvrPiz-t, AVR-Pita and Avr-Pi9 have been cloned molecularly [26,30–35]. They are all translocated into host cells and do not show similarities to proteins of known function with the exception of AVR-Pita that shows homology to neutral zinc proteases [6]. For 7 of them, the matching rice immune receptors that are in all cases cytoplasmic nucleotide-binding and leucine-rich repeat domain proteins (NLRs) have been identified [36–41].

In the present study, the 3-dimensional structures of the *M*. *oryzae* effectors AVR-Pia and AVR1-CO39 were investigated to deepen our understanding of fungal effector function and diversity. NMR analysis revealed that the structures of both proteins consist of two anti-parallel β-sheets, each having three strands, and linked by one disulfide bond Structural similarity searches revealed that the *M*. *oryzae* effector AvrPiz-t and the effector ToxB from the wheat pathogen *Pyrenophora tritici-repens* have similar 6 β-sandwich structures with the same topology [42,43]. Comparisons of the structures of the four effectors that we named MAX-effectors revealed that they share a common architecture but no sequence consensus. Structure-informed and pattern-based searches identified large numbers of weakly homologous MAX-effector candidates in *M*. *oryzae* and *M*. *grisea*, and limited numbers or no homologs in other phytopathogenic ascomycete fungi. Expression profiling indicated that the majority of the *M*. *oryzae* MAX-effector candidates are expressed during early infection. MAX-effectors therefore seem to have undergone a lineage-specific expansion in the *Pyricularia* genus that may be driven by duplications and rapid adaptation to new functions involving important changes of surface properties but conservation of protein architecture. This evolutionary process has the potential to generate large families of structurally related proteins without sequence similarity and may serve as a paradigm for effector evolution and diversification in phytopathogenic ascomycete fungi.

## Results

### Protein expression

AVR1-CO39 and AVR-Pia proteins, deleted for their endogenous secretion signal, were expressed in *E*. *coli* with an N-terminally fused signal peptide for secretion in the bacterial periplasm that is cleaved upon secretion, an N-terminal His_6_-tag for purification and a TEV1 cleavage site. Recombinant proteins were soluble and were purified to homogeneity from periplasmic protein extracts by Ni-agarose affinity and gel exclusion chromatography (S1 Fig). Both recombinant Avr proteins eluted as monomers from gel exclusion chromatography.

### NMR analysis

**Fig 1:**
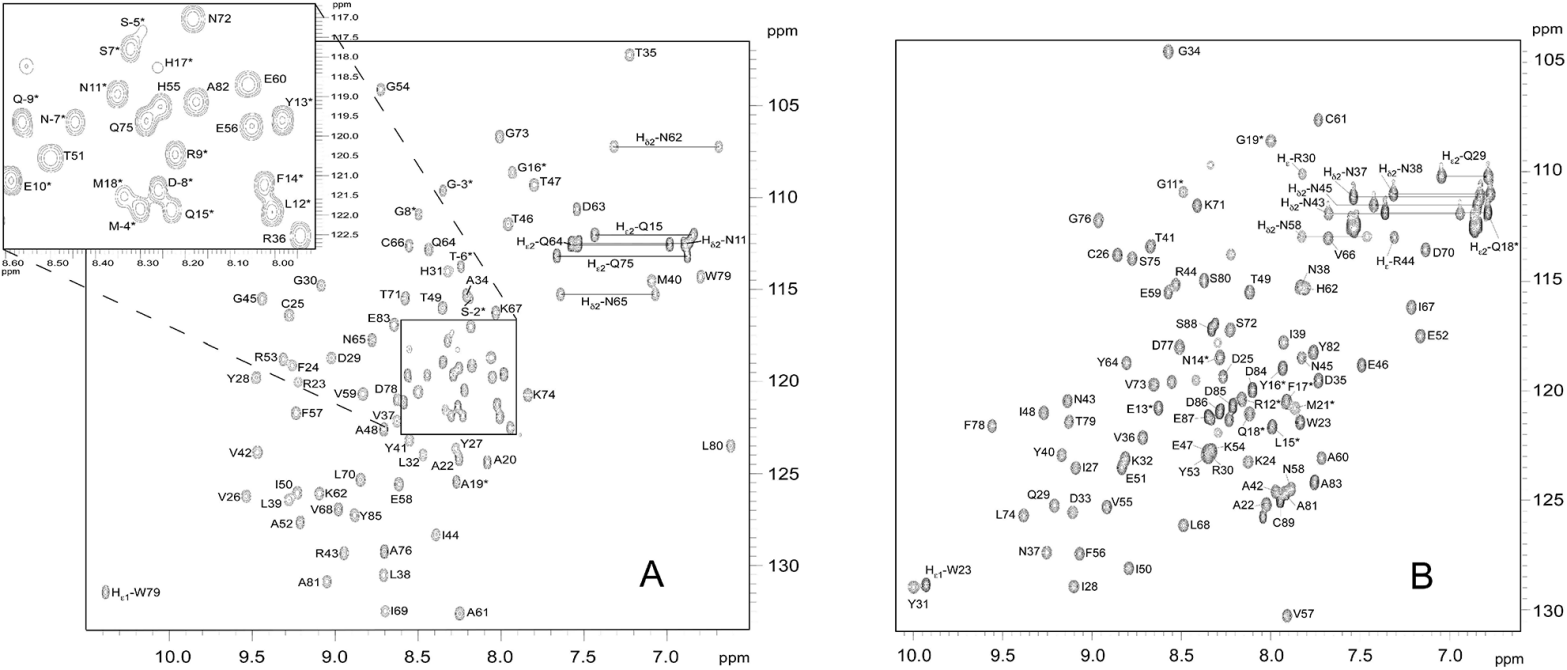
^15^N-HSQC spectra of (A) AVR-Pia and (B) AVR1-CO39. In the 15N-HSQC spectra of (A) AVR-Pia and (B) AVR1-CO39, each peak comes from N-H chemical connectivity and has ^15^N and ^1^H chemical shift coordinates. There is one backbone N-H group per residue, leading to one HSQC peak per residue. A side-chain NH_2_ group gives two HSQC peaks with one common N coordinate. Other side-chains NH groups may also be observed, as Trp Nε1-H and Arg Nε-H. (*) indicates residues in the N-terminal tail. The mature proteins start at residue Ala20 and Trp23 for AVR-Pia and AVR1-CO39, respectively [26,32]. The NH_2_ side chains resonances were assigned. The resonances of the tryptophan indole groups are specifically labelled Hε1.

Recombinant, ^15^N and ^13^C-labelled AVR1-CO39 and AVR-Pia proteins produced in ^15^N and ^13^C-labelled minimal medium were used for structure determination by two- and three-dimensional NMR experiments. Three-dimensional (3D) HNCO, HNCA, HN(CO)CACB, HN(CA)CO, HNCACB, 2D ^13^C-detected CON, CACO and 2D-COSY-DQF(D_2_O) and TOCSY(D_2_O) experiments were used for the backbone and aliphatic side chain resonance assignments. 3D ^15^N-edited NOESY-HSQC and 2D-NOESY(D_2_O) spectra were collected to confirm the chemical shift assignments and generate distance restraints for structure calculations. (Fig 1 and S1 Table). The assigned ^1^H,^15^N-HSQC spectra were well dispersed. Residues from the N-terminal tags are still resolved. All amino acids of AVR-Pia and almost all of AVR1-CO39 have {^1^H-^15^N} NOE values above 0.8 indicating highly defined structures with low flexibility (S2 Fig). Only N-terminal tags, below residue number 22–23, and C-terminal sequences of AVR1-C039 (amino acids 80–89) show increased flexibility. The strong dα_N_(i, i+1) NOEs and weak d_NN_(i, i+1) NOEs are indicative of a β-structure and consistent with the six β-strands observed in AVR-Pia and AVR1-CO39 (S3 Fig). NHs in slow exchange were consistent with hydrogen bonding networks and were used to derive constraints for the structure calculations.

The ratios of R_2_ to R_1_ relaxation rates of AVR-Pia and AVR1-CO39 were consistent with a monomeric molecular size (AVR-Pia τ_c_ = 6.2 ± 0.3 ns and AVR1-CO39 τ_c_ = 5.7 ± 0.4 ns) and thus confirm that both Avrs form monomers in solution (S1 Table) [44].

### AVR-Pia and AVR1-CO39 have similar β-sandwich structures

**Fig 2:**
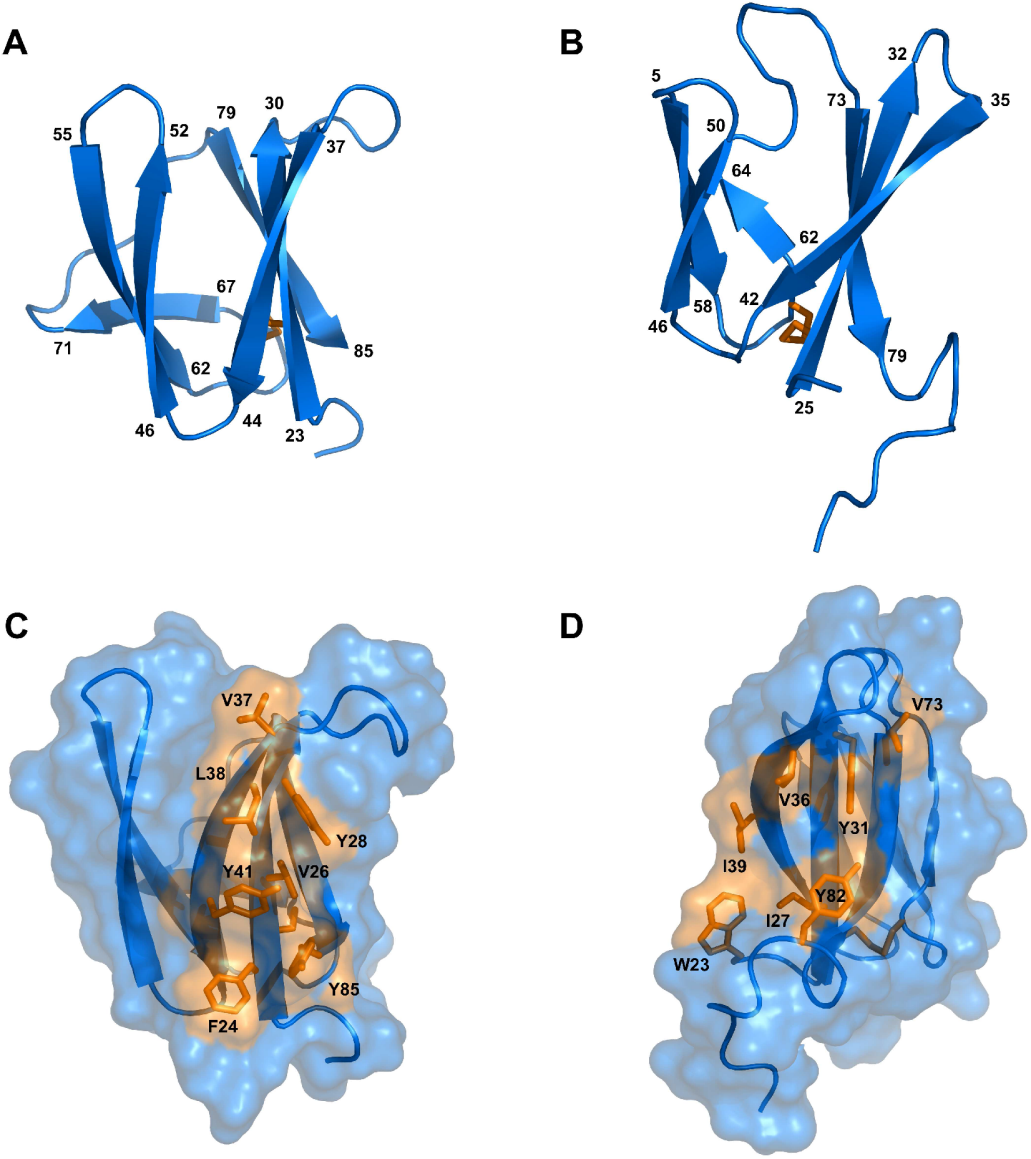
Solution structures of mature AVR-Pia and AVR1-CO39. Cartoon representations of AVR-Pia (A) and (B) AVR1-CO39 highlight the similar β-sandwich structure of both proteins. Yellow sticks represent disulfide bonds. Numbers indicate the residues at β-strands borders. A surface view reveals extended hydrophobic patches on one of the surfaces of AVR-Pia (C) and AVR1-CO39 (D) that are composed of exposed hydrophobic residues labelled in yellow. The Figs were generated using PyMOL (http://www.pymol.org).

The solution structures of AVR-Pia and AVR1-CO39 were determined based on 1541 and 1286 NOE-derived distance restraints, 90 and 72 dihedral angle restraints and 20 and 15 hydrogen bond restraints, respectively (Fig 2 and Table 1 and S4 Fig). A disulfide bridge between Cys25-Cys66 for AVR-Pia and between Cys26-Cys61 for AVR1-CO39 was added based on cysteine ^13^Cβ chemical shifts and DTNB quantification of free thiols. The Pro65 in AVR1-CO39 has been determined to be in a cis-conformation according to the ^13^Cβ chemical shift at 34.4 ppm and strong sequential Hα-Hα NOE. The best conformers with the lowest energies, which exhibited no obvious NOE violations and no dihedral violations > 2° were selected for final analysis.

**Table 1:** Statistics for 20 NMR structures of AVR-Pia and AVR1-CO39.

	AVR-Pia	AVR1-CO39
**NOE restraints**	1541	1286
Short range (|i-j|≤1)	1022	745
Medium range (1<|i-j|<5)	128	157
Long range (|i-j|≥5)	391	384
Dihedral restraints (a)	90	72
**Number of NOE violations**		
> 0.1 Å	9.30 ± 1.29	22.45 ± 2.76
> 0.2 Å	0.35 ± 0.47	0.60 ± 0.74
> 0.3 Å	0.05 ± 0.01	0
> 0.4 Å	0	0
**Dihedral violations**		
> 2°	0.15 ± 0.26	0.05 ± 0.10
> 4°	0	0
**Ramachandran plot statistics** **(b)**		
most favorable regions (%)	86.2	83.2
additionally allowed regions (%)	13.1	15.4
generously allowed regions (%)	0	1.3
disallowed regions (%)	0.7	0.3
**Pairwise RMSD (Å)** **(c)**		
Backbone	0.72 ± 0.14	0.49 ± 0.11
Heavy atoms	1.34 ± 0.18	1.02 ± 0.14

Structures were calculated using CYANA, refined using CNS, and analyzed using PROCHECK.

(a) Residues in regular secondary structures were derived from the chemical shifts using TALOS+ software.

(b) PROCHECK was used over the residues 24–85 for AVR-Pia and over the residues 23–83 for AVR1-CO39.

(c) Main chain atoms (N, Cα, C) over the residues 24–85 for AVR-Pia and over the residues 23–83 for AVR1-CO39.

Surprisingly, both AVR-Pia and AVR1-CO39 proved to possess the same secondary structure elements arranged with the same topology in similar three-dimensional structures (Fig 2). Both proteins are composed of 6 β-strands that form two antiparallel β-sheets packed face-to-face and connected by loops (Fig 2). The first sheet is formed by the three β-strands β1, β2 and β6 while the second sheet contains β3, β4 and β5. In both cases, the two β-sheets pack together by an internal core of hydrophobic residues and one disulfide bridge and the structures belong to the β-sandwich classification. In both Avrs, the β-strands overlay and are similarly oriented (vide infra) but loops differ in length and structure.

### AVR-Pia and AVR1-CO39 possess a hydrophobic surface patch

The surface properties of AVR-Pia and AVR1-CO39 are different with the exception of a hydrophobic patch located in both proteins on the side of the β-sandwich that is formed by the first β-sheet (β1-β2-β6) (Fig 2C and 2D). In AVR-Pia, this solvent exposed hydrophobic surface is constituted by the residues F24, V26 and Y28 in β1, V37, L38 and Y41 in β2, and Y85 in β6, and has an area of 372 Å^2^. In AVR1-CO39, the solvent exposed hydrophobic surface of the first β-sheet is formed by the residues I27 and Y31 in β1, V36 and I39 in β2 and V73 in β6, as well as W23 from the N-terminus and Y82 from the C-terminus, and has a surface area of 280 Å^2^.

### ToxB and AvrPiz-t are structural homologs of AVR1-CO39 and AVR-Pia

**Fig 3:**
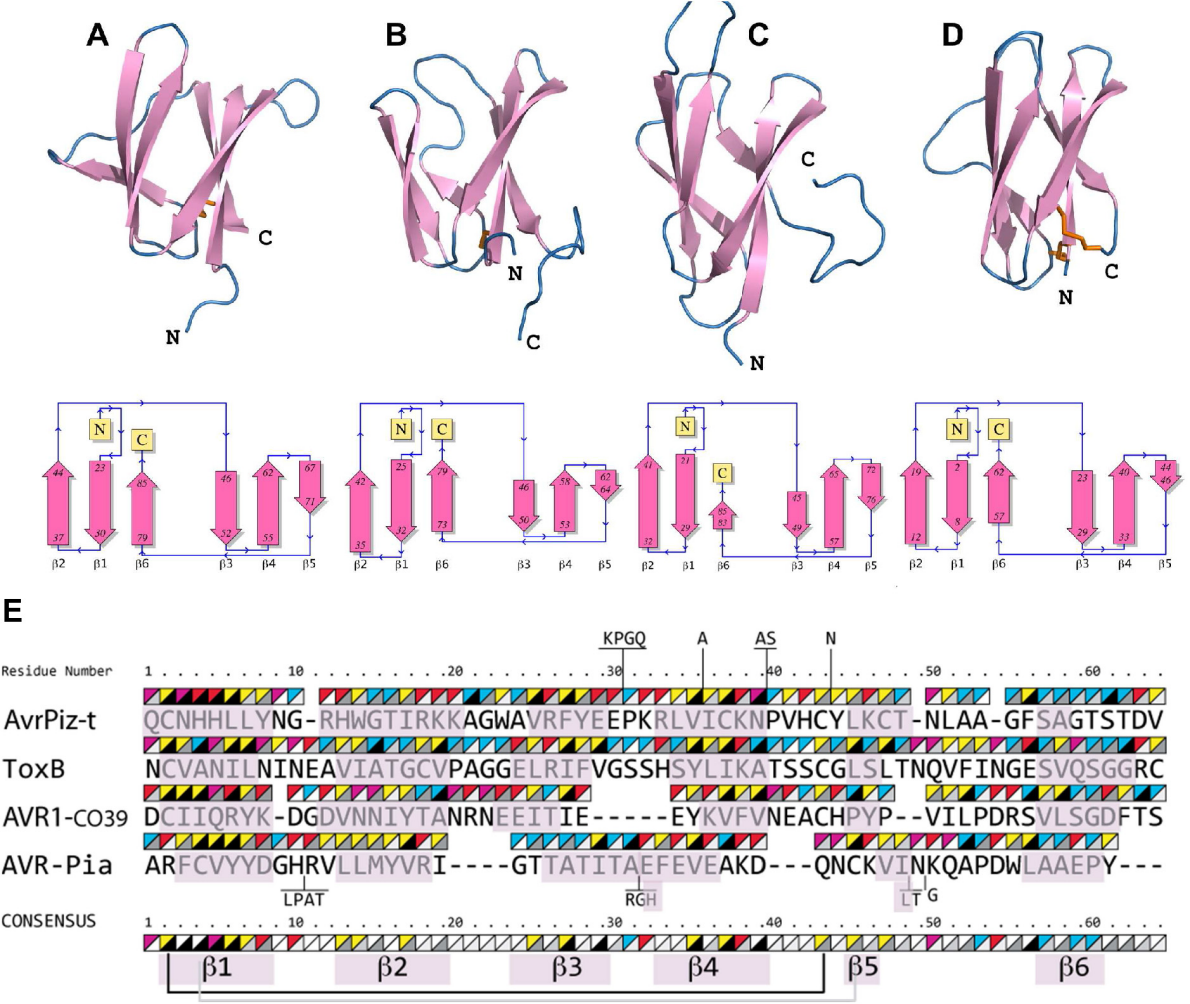
AVR-Pia, AVR1-CO39, AvrPiz-t and ToxB have similar 6 β-sandwich structures. Topology diagrams (lower row) show that AVR-Pia (A), AVR1-CO39 (B), AvrPiz-t (C) and ToxB (D) possess the same fold. Ribbon diagrams (upper row, generated with PyMOL (http://www.pymol.org)) highlight similarities of their structures. Disulfide bonds are shown in the ribbon diagrams by orange sticks. All four structures were superimposed and a structural alignment was derived using DALI with the ToxB sequence as the reference for numbering (E). Residues not aligned to ToxB are connected by vertical lines and correspond to insertions in loops of AvrPiz-t and AVR-Pia. Triangles over the residues indicate chemical properties (upper-left triangle: yellow for hydrophobic, red for charged, pink for Asn and Gln and blue for other residues) and solvent accessibility (lower-right triangle: from black for buried to white for solvent-exposed). The consensus is defined by at least three similar residues per position. Residues forming β-strands are pink. Disulfide bridges in AVR1-CO39 and ToxB are shown below the consensus by a black line and for AVR-Pia by a grey line. For AvrPiz-t, no disulfide bridge was reported despite presence of the two conserved cysteins [42].

To identify structural homologs of AVR-Pia and AVR1-CO39, structural similarity searches were performed using the Dali server and the Protein Data Bank [45]. Both queries, with AVR1-CO39 and AVR-Pia, identified the secreted effector protein ToxB from the wheat tan spot pathogen *Pyrenophora tritici-repentis* as well as its natural allele Toxb as the closest structural homologs with the highest Z-scores (S2 Table and Fig 3) [43]. Like, AVR-Pia and AVR1-CO39, ToxB is secreted during infection and is an important determinant of virulence for the tan spot fungus [46]. In addition, the search with AVR1-CO39 identified AvrPiz-t, another avirulence effector of *M*. *oryzae* that is sequence-unrelated to AVR-Pia and AVR1-CO39 but structurally similar [42]. A pairwise similarity matrix using root-mean-square deviation (rmsd, measured in Å) and DALI Z-scores [45] was established revealing that all proteins are structurally related and that ToxB is closer to all other three structures than the others among them (S2 Table). ToxB and AvrPiz-t are like AVR-Pia and AVR1-CO39, composed of two three-stranded antiparallel β-sheets, β1-β2-β6 and β3-β4-β5, forming a six β-sandwich (Fig 3A–3D). Structure-based sequence alignments provided by DALI revealed, at a first glance, no obvious conservation, but also no clear consensus except buried hydrophobic residues alternating with exposed polar amino acids in the β-strands (Fig 3E). The β-strands β1 and β2 are very similar in length and position in all four proteins, while β3, β4 and β6 display more variation. β5 is the shortest and the most irregular strand. As expected for β-strands, buried and exposed residues alternate, with the exception of β1 where residues have a tendency to be more buried. This is due to the packing of β1 in between the β2 and β6 strands. The loops connecting the β-strands have variable length, and are the sites where most of the residue insertions occur. The disulfide bond between C2 and C43 (ToxB numbering) is well conserved but shifted “in phase” by two residues in AVR-Pia (Fig 3E).

### Psi-Blast searches identify in *M*. *oryzae* and *M*. *grisea* multiple effector candidates with similarities to *Magnaporthe* Avrs and ToxB

The unexpected finding, that all three *M*. *oryzae* effectors that have been characterized for their structure so far and one effector from an only very distantly related fungal group are structurally related raised the possibility that these four effectors are members of a widely distributed and abundant fungal effector family characterized by a common β-sandwich structure and high sequence divergence. Simple Blast searches are not suited to identify such distantly related proteins and when performed with the protein sequence of effectors from ascomycete fungi, generally identify no or only very few conserved homologs in the same species. Therefore, more sensitive Psi-Blast searches that use position-specific scoring matrices were performed with AVR-Pia, AVR1-CO39, AvrPiz-t and ToxB. The searches were performed on a protein sequence database combining the protein sequences of the *M*. *oryzae* reference isolate 70–15, of 5 other rice-infecting *M*. *oryzae* isolates (TH16, TH12, PH14, FR13 and Guy11), three *M*. *oryzae* isolates with other host specificities (BR32, US71 and CD156 specific for wheat, *Setaria italica* and *Eleusine coracana*) and one isolate of the sister species *M*. *grisea* (BR29). These additional *M*. *oryzae* and *M*. *grisea* protein sequences were obtained by whole genome re-sequencing and de novo annotation of proteins and are accessible at http://genome.jouy.inra.fr/gemo [47]). After 4 Psi-Blast iterations and filtering of the results for sequences having an alignment length of at least 40 residues, an overall protein size of less than 180 amino acids and the presence of a predicted signal peptide, 3, 8 and 4 homologs of AVR-Pia, 16, 25 and 16 homologs of AVR1-CO39 and 5, 9 and 6 homologs of ToxB were detected in respectively 70–15, TH16 and BR29 (S3 Table, orthologous sequences present in 70–15 and TH16 were only counted for 70–15). For the other *M*. *oryzae* isolates similar numbers of homologs as in TH16 were found. The elevated number of homologs present in these isolates but not in 70–15 are due to the fact that the pipeline used for protein annotation in the re-sequenced genomes identified many additional small secreted proteins that are not annotated in 70–15 although the corresponding coding sequences are present in its genome [47]). The similarities were weak (frequently less than 25% identity) but they were consistent with the structural alignment (Fig 3) and included the two cysteine residues. For AvrPiz-t, no homologs that were not already identified by standard Blast were identified in the Psi-Blast search. When 25 additional fungal genomes, including the closely related fungi *M*. *poae* and *Gaeumannomyces graminis* were added to the database for the Psi-Blast searches, only very limited numbers of homologs (0, 1 or 2) with frequently low e-value scores were identified in other fungi. This suggested that effectors with similaritiy to ***M*** *agnaporthe* **A**vrs and To**x**B named in the following MAX-effectors that potentially also have 6 β-sandwich structure are present with low frequency in other fungal pathogens but were strongly amplified and diversified in *M*. *oryzae* and *M*. *grisea* that both belong to the genus *Pyricularia* in the *Pyriculariae* family [48].

### HMM searches identify a huge MAX-effector family in *M*. *oryzae* and *M*. *grisea*

To exclude that the Psi-Blast search missed MAX-effectors in the additional fungal genomes due to biases in the search matrix or too low sensitivity and to deepen the search for this class of effectors in *M*. *oryzae* and *M*. *grisea* genomes, a hidden Markov model (HMM)-based profile search was performed. This type of profile search is among the most powerful procedure for detecting with high accuracy remote homologies between proteins.

As a first step, a high stringency Blast search with the three *M*. *oryzae* effectors and a Psi-Blast search with ToxB was performed and the resulting set of closely related sequences was aligned in a multiple sequence alignment constrained by the structural alignment of AVR-Pia, AVR1-CO39, AvrPiz-t and ToxB (S5A Fig). For the *M*. *oryzae* effectors, the Blast search identified orthologs of the effectors with few polymorphisms in different *M*. *oryzae* isolates. In addition, for each *M*. *oryzae* effector, one paralog was identified in *M*. *oryzae* and one or two paralogs were identified in the *M*. *grisea* isolate BR29 (S5B Fig). For the *M*. *oryzae* paralogs, generally several different alleles were identified. For ToxB, in addition to highly homologous sequences from *P*. *tritici-repentis* and *P*. *bromi*, 1 homolog was identified in *M*. *oryzae*, *Bipolaris oryzae* and *Colletotrichum higginsianum*, 2 in C. *fioriniae*, 3 in *C*. *orbiculare* and 4 in *C*. *gloeosporioides*. (S5B Fig).

As a second step, an HMM profile was built, starting from the structure-guided multiple sequence alignment from step1 (S5A Fig) and by iteratively searching for homologs in a database containing the small secreted proteins (<170 amino acids) of 25 pathogenic and non-pathogenic ascomycete fungi and of the 9 re-sequenced *M*. *oryzae* and *M*. *grisea* isolates from which completely redundant sequences had been removed. At each iteration, the recovered sequences were filtered for alignment of the two cysteins with a spacing of 34 to 49 amino acids and used to generate a new profile used in the next iteration. The interval of 34 to 49 amino acids was fixed, based on the frequencies of cystein spacings in HMM searches run without this constraint.

This search recovered 161 new, more distantly related sequences of which 154 were from *M*. *oryzae* or *M*. *grisea*, 5 from 3 different *Colletotrichum* species, 1 from *Lepthosphaeria maculans* and 1 from *Mycosphaerella graminicola* (recently renamed *Zymoseptoria tritici*) (S6A Fig). This suggests that MAX-effectors have been massively and specifically expanded in *M*. *oryzae* and *M*. *grisea*. However, it also indicates their presence in other fungal species, i. e. in *Colletrichum* spp. where they seem to occur at elevated frequencies. The alignment and clustering of the set of 200 sequences combining the 39 sequences used for the initial profile and the 161 new sequences revealed clusters of orthologous sequences originating from the different *M*. *oryzae* isolates with weak sequence polymorphism between orthologs (S6A and S6B Fig). Frequently, orthologs of *M*. *oryzae* can be identified in *M*. *grisea* but never in other fungi. Sequences from different orthologous clusters have high sequence diversity. Only in 3 cases, statistically significant clusters, supported by bootstrap values bigger than 50% can be identified that contain 2 distantly related MAX-effectors or MAX-effector clusters of *M*. *oryzae*.

**Fig 4:**
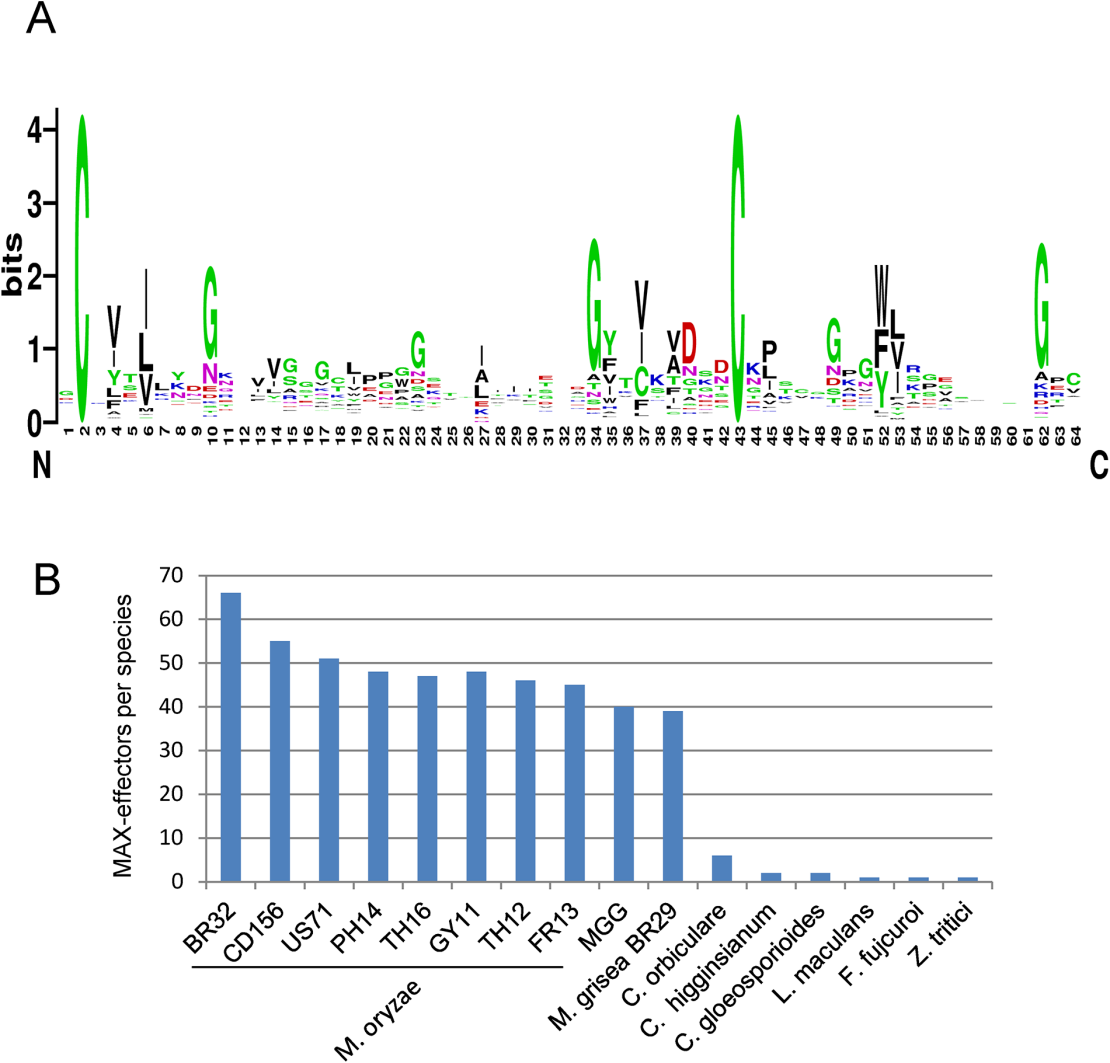
Large numbers of MAX-effectors sharing a characteristic sequence pattern are present in *M*. *oryzae* and *M*. *grisea*. A) Sequence pattern of MAX-effectors. The sequence logo was generated using the alignment of MAX-effector candidates identified by a high stringency HMM search (S6 Fig). (B) Numbers of MAX-effector candidates detected by a low stringency HMM sequence pattern search. A database combining 25 pathogenic and non-pathogenic ascomycete fungi and 9 *M*. *oryzae* and *M*. *grisea* isolates was searched with an HMM pattern based on a structural alignment of AVR-Pia, AVR1-CO39, AVR-Pia and AvrPiz-t.

A sequence logo derived from the multiple alignment shows the invariant cysteine residues (position 2 and 43 in mature ToxB) that constitute the alignment framework, as well as additional positions that are specifically enriched (Fig 4A). There is a propensity for hydrophobic residues in positions 4 and 6, corresponding to hydrophobic positions in strand β1, in position 27, corresponding to a hydrophobic residue in β3 and in positions 35, 37 and 39 corresponding to β4. Positions 10, 23, 40 and 49 are in loop regions between the pairs of strands β1-β2, β2-β3, β4-β5 and β5-β6 respectively, and are enriched in glycine, polar or charged residues.

The resulting HMM profile was used to search with a relaxed cut-off two different databases: (i) the UniRef90 database that contains non-redundant sequences from a wide range of different organisms and that was used to determine in which type of organisms proteins with the MAX-effector motif occur and to evaluate by this the specificity of the motif and (ii) the previously described fungal genomes and *M*. *oryzae* and *M*. *grisea* database to get a precise view of the occurrence of MAX-effectors in a broad range of ascomycete fungi.

The search of the UniRef90 database recovered 70 sequences. All but 3 were from phytopathogenic ascomycete fungi (S7A Fig). The exceptions were from a bacteria, *Pseudomonas* sp. *StFLB209*, living in association with plants, from tomato (*Solanum lycopersicum*) and from a nematode-parasitic fungus (*Arthrobotrys oligospora*) and had low e-values. Among the fungal sequences, 49 were from *M*. *oryzae* and included AVR1-CO39 and AVR-Pia. The remaining 18 corresponded to previously identified effectors from *Colletotrichum* species (5 *C*. *orbiculare*, 2 *C*. *higgensianum*, 3 *C*. *gloeosporioides*, 2 *C*. *fioriniae*) that belong as *M*. *oryzae* to the class of Sordariomycetes and *Z*. *tritici*, *L*. *maculans* and *B*. *oryzae* as well as ToxB from *P*. *tritici-repentis* and *P*. *bromi* that are all from the class of Dothideomycete fungi. Clustering of the sequences revealed high sequence diversity and, apart from the Tox-B cluster, no or extremely limited relatedness could be identified (S7A and S7B Fig). Interestingly, with slightly different settings, this search also recovered the well characterized AVR-Pik effector from *M*. *oryzae* [26]. AVR-Pik clearly fits the MAX-effector pattern but was discarded in the other searches since its secretion signal is not recognized by the SignalP4.1 program used for filtering of the results.

The search of the previously described *Magnaporthe* and other fungal genomes database not filtered for redundancy recovered only limited numbers of MAX-effectors in non-*Magnaporthe* fungal genomes that had, with the exception of one effector from *Fusarium fujicuroi*, already been retrieved in the other searches (Fig 4B and S8A Fig). In *M*. *oryzae*, between 67 and 38 MAX-effectors per isolate were identified while in *M*. *grisea*, 37 MAX-effectors were identified (Fig 4B). 46 of the 55 MAX-effectors identified by Psi-Blast in *M*. *oryzae* 70–15 and TH16 and in *M*. *grisea* BR29 (S3 Table) were also found by this HMM search. Alignment and clustering shows that the *M*. *oryzae* MAX-effectors are generally present in the majority of *M*. *oryzae* isolates and are grouped in clusters of orthologs (S8A and S8B Fig). Many of these orthologous clusters also contain an ortholog from the *M*. *grisea* isolate BR29 that shows however higher sequence divergence. Only six statistically significant clusters (bootstrap > 50%) that contain more distantly related *M*. *oryzae* effectors from different orthologous groups are identified. Otherwise, the sequence diversity between proteins from different *M*. *oryzae* ortholog clusters is so strong that classical tree building methods do not detect statistically significant sequence relatedness. The non-*Magnaporthe* MAX-effectors do not cluster significantly with *Magnaporthe* MAX-effectors and 8 of the 10 *Colletotrichum* effectors are comprised in three different *Colletotrichum*-specific clusters.

Taken together, the different HMM searches reveal that the MAX-effector motif is specific for effectors from phytopathogenic ascomycete fungi. MAX-effectors are identified with low frequencies in phytopthogenic ascomycete fungi from the class of Dothideomycetes and seem to have expanded moderately in different *Colletotrichum* species (i.e. *Colletotrichum orbiculare*). Only in *M*. *oryzae* and *M*. *grisea*, MAX-effectors expanded and diversified massively to become a dominating family of virulence effectors in these pathogens.

### Expression profiling shows that a majority of MAX-effectors is expressed specifically during biotrophic infection

**Fig 5:**
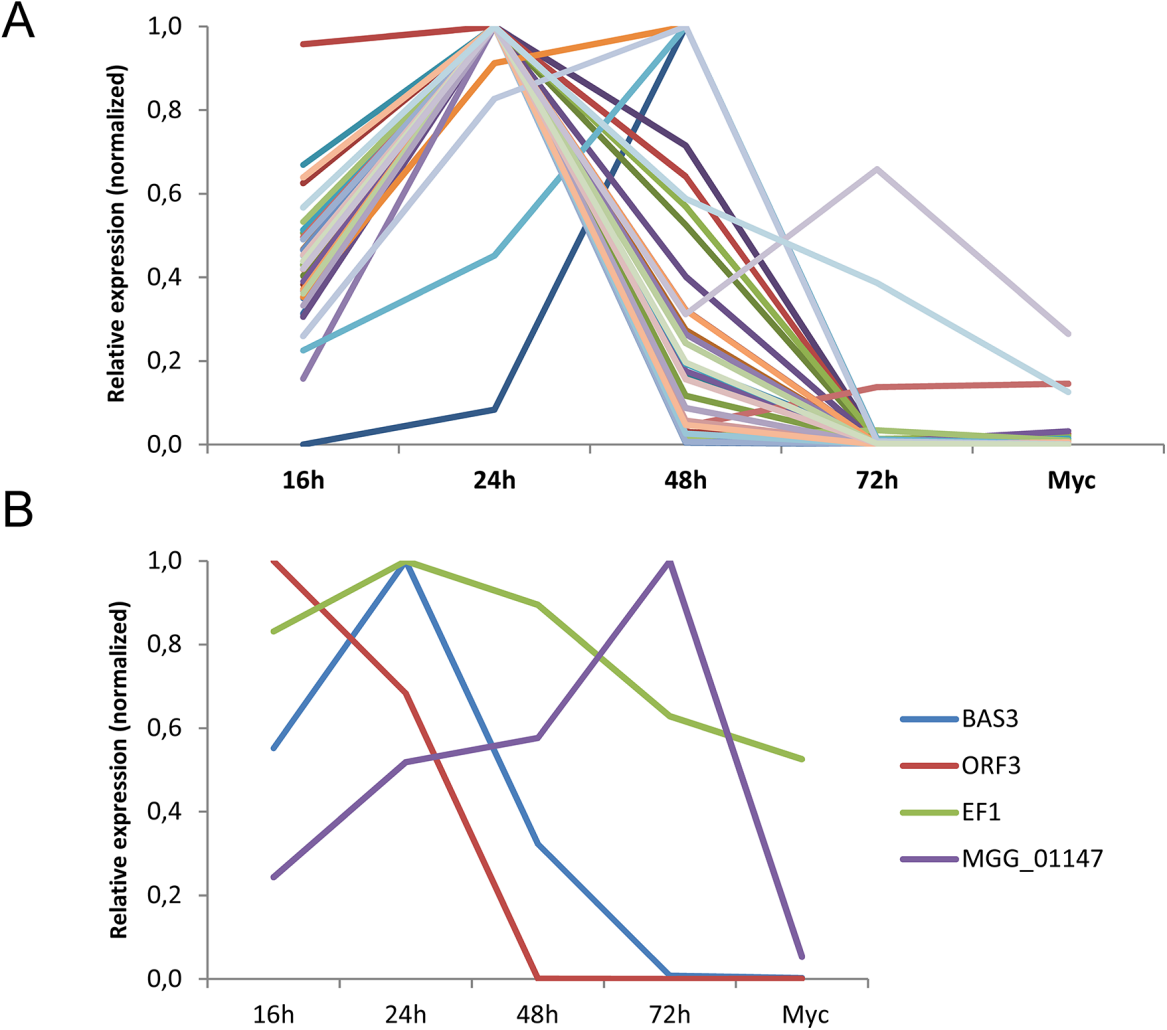
The majority of *M*. *oryzae* MAX-effectors is expressed specifically during biotrophic infection. mRNA levels of *M*. *oryzae* genes coding for 32 different MAX-effectors (A) and marker genes (B) for appressorium formation and very early infection (*ORF3* of the *ACE1* cluster, *MGG_0838*1), biotrophic infection (*BAS3*, *MGG_11610*), late infection *(MGG_01147*) and constitutive expression (EF1α, *MGG_03641*) were determined by q-RT-PCR in rice leaf samples harvested 16, 24, 48 and 72 h after inoculation and mycelium grown *in vitro*. Relative expression levels were calculated by using expression of a constitutively expressed *Actin* gene (*MGG_03982*) as a reference and normalized with respect to the highest expression value. Values are means calculated from the relative expression values of three independent biological samples. Individual expression profiles are in S9 Fig.

To test if the *M*. *oryzae* MAX-effectors identified by the HMM profile search could be involved in plant infection, the expression of 50 different candidate MAX-effector-coding genes was analyzed by qRT-PCR in infected rice leafs and in *in vitro* grown mycelium (S4 Table). 30 genes showed early infection-specific expression with a majority of profiles (25) that strongly resemble the biotrophy effector marker gene *BAS3* (Fig 5 and S9A and S9D Fig) [23]. The expression pattern of all these genes and of 3 genes coding for MAX-effectors identified only by Psi-Blast searches was clearly different from the markers of very early or late infection (Orf3 and MGG01147, respectively). For 18 genes, no significant expression was detected and only 2 genes were expressed constitutively with significant expression in the mycelium (Fig 5 and S9C and S9D Fig). Therefore, the majority of the MAX-effector candidates seems specifically expressed during biotrophic infection and can therefore be considered as potential virulence effectors.

## Discussion

In this study, we have determined by NMR spectroscopy the 3-dimensional structures of two different effectors of *M*. *oryzae*, AVR1-CO39 and AVR-Pia. Although the two proteins have no evident sequence similarity they possess similar 6 β-sandwich structures formed in both cases by two β-sheets each formed of three β-strands oriented in an antiparallel manner. Interestingly, similar β-sandwich structures have previously been found for AvrPiz-t, the only other structurally characterized *M*. *oryzae* effector and for ToxB, an effector from an only very distantly related plant pathogenic ascomycete fungus, *P*. *tritici-repentis*. Overlay of the structures and structural alignments revealed that the nature and number of secondary structural elements and the topology of their fold are the same in all four effectors. In addition, all four proteins are stabilized by buried hydrophobic residues coming for their majority from the β-strands and by a disulfide bond between conserved cysteins located in the beginning of β1 and in the beginning or just before β5. However, the orientation and the length of certain β-strands, i.e. β-5, vary considerably and the sequences and the length of loops are highly variable resulting in proteins with very different shapes and surface properties. Due to the high sequence diversity, similarity among the MAX-effectors is therefore only detected when their structure is taken into consideration.

### Hydrophic surface patches in AVR-Pia and AVR1-CO39 are potential sites of protein-protein interaction

The only similarity of the surfaces of AVR1-CO39 and AVR-Pia is an extended hydrophobic area on the surface formed by β1, β2 and β6. Such extended and exposed hydrophobic areas are uncommon since protein surfaces are generally in contact with solvent water molecules and they are frequently involved in protein-protein interactions. Previous studies on the recognition of AVR-Pia by the rice NLR immune receptor RGA5 support that the hydrophobic surface of AVR-Pia could indeed be involved in protein binding [37]. AVR-Pia binds physically to a C-terminal domain of RGA5 homologous to heavy metal-associated (HMA) domain proteins related to the copper chaperone ATX1 from *Saccharomyces cerevisiae* (RATX1 domain). This binding is required to derepress a second NLR RGA4 that activates resistance signaling [49]. A natural allele of AVR-Pia (AVR-Pi-H3) where the surface exposed phenylalanine 24 and threonine 46 situated respectively in and at the border of the hydrophobic patch are replaced by serine and asparagine loses binding to RGA5_RATX1_ and does not trigger resistance [37]. Structural information will now guide further functional studies to elucidate if other amino acids situated in or at the border of the hydrophobic patch are also involved in RGA5_RATX1_-binding and to validate by this the role of the hydrophobic patch as a protein-protein interaction surface.

### MAX-effectors have different molecular properties and activities

Common features of the *M*. *oryzae* MAX-effectors are that they act intracellular in host cells [21,24,32] and are recognized by NLR immune receptors in resistant rice genotypes: AVR1-CO39 and AVR-Pia by the same NLR pair RGA4/RGA5 and AvrPiz-t by the NLR immune receptor Piz-t [37,39,41]. While the molecular bases of the recognition of AVR1-CO39 and AVR-Pia by RGA4/RGA5 are beginning to be elucidated, details of AvrPiz-t recognition are not known. Also, whether the three *M*. *oryzae* MAX-effectors target similar host processes and host proteins is not known. AvrPiz-t was described to target the host ubiquitin proteasome system by binding and inactivating the RING E3 ubiquitin ligase APIP6 [21] but virulence targets of AVR-Pia and AVR1-CO39 have not been described. However, it has been hypothesized that both proteins target RATX1 proteins homologous to the RGA5_RATX1_ domain that was suggested to act as a mimic for AVR-Pia and AVR1-CO39 targets [50]. Therefore, we assume that AvrPiz-t on the one hand and AVR-Pia and AVR1-CO39 on the other have different molecular activities and target different host proteins. This would be in accordance with the high divergence of their shapes and their surface properties. That AVR-Pia and AVR1-CO39 interact with the same immune receptor by binding to the same sensor domain and potentially interact with the same host targets is striking because apart from the extended hydrophobic patch on the β1β2β6 surface they share no apparent similarities with respect to their shapes and surfaces. It will therefore be important to elucidate in the future which amino acids of AVR-Pia and AVR1-CO39 bind to RGA5_RATX1_ and which surfaces of RGA5_RATX1_ are involved in binding to each of the two effectors to better understand specificity in effector recognition. In addition, identification of AVR1-CO39 and AVR-Pia targets as well as ToxB targets for which molecular details of activity are also lacking will be important to understand how MAX-effectors promote virulence and to understand the link between MAX-effector structure and function.

### MAX-effectors are a highly diversified effector family specific to phytopathogenic ascomycete fungi and underwent expansion in *M*. *oryzae* and *M*. *grisea*

Structure-informed pattern searches identified huge numbers of MAX-effector candidates that possess as the structurally characterized MAX-effectors very high sequence diversity and probably also possess a 6 β-sandwich structure stabilized by buried hydrophobic residues from β-strands and a disulfide bond between conserved cysteins connecting β1 and β5. Systematic prediction of the secondary structure of the MAX effector candidates using SSPRO 5 software identified with high frequency two β-strands, β1 located after the first cysteine and β4 located before the second cysteine (S10 Fig). The other regions of the sequences had more variable secondary structure predictions which is also reflected by a less defined pattern in these regions (Fig 4A). High sequence diversity among MAX-effector candidates could as in the case of the structurally characterized MAX-effector be the consequence of interchangeability of buried hydrophobic core residues, variation in the lengths of some β-strands (i.e. β5), exchange of surface exposed residues and deletion or insertion of residues in exposed loops.

MAX-effectors were specifically detected in phytopathogenic ascomycetes from the classes of Sordariomycetes and Dothideomycetes. One MAX-effector per species was detected in phytopathogenic fungi of the class of Dothideomycetes (*L*. *maculans*, *P*. *tritici-repentis*, *Z*. *tritici* and *B*. *oryzae*) and higher numbers (2–6) occur in fungi from the genus *Colletotrichum*. Only in *M*. *oryzae* and *M*. *grisea* that are both from the genus *Pyricularia* huge numbers of MAX-effector candidates were detected and expression profiling confirmed that most of them are likely *bona fide* effectors expressed specifically during biotrophic early infection. With 40–60 effectors which represents 5–10% of the candidate effectors of individual *M*. *oryzae* or *M*. *grisea* isolates, MAX-effectors can be considered a dominant class of effectors in these fungi [24,47]. This is further supported by the finding that 5 of the 51 biotrophy-associated proteins identified by transcriptome analysis are MAX effectors (MG02546, MG08414, MG08482, MG09425 and MG09675) [23]. Also, the *M*. *oryzae* effector AVR-Pik fits the MAX-effector pattern further highlighting the outstanding importance of this effector family that comprises 4 out of 8 cloned Avr effectors in the blast fungus [6]. It is striking that the only other group of fungi with elevated numbers of MAX effectors are *Colletotrichum* species. *Colletotrichum* fungi are phylogenetically only distantly related to *M*. *oryzae* and *M grisea* but employ a similar hemibiotrophic infection strategy characterized by appressorium-mediated penetration into the host and growth inside invaded plant cells during biotrophic infection. It will be interesting to determine in the future whether MAX effectors play similar roles in these early infection processes in both groups of fungi.

In *Gaeumannomyces graminis* and *M*. *poae* that belong to the closely related *Magnaporthaceae* family no MAX-effectors were detected [48]. The expansion of MAX-effectors therefore occurred probably in a common ancestor of *M*. *oryzae* and *M*. *grisea* since clear orthologous relations can be established between many MAX-effectors from *M*. *oryzae* and *M*. *grisea* but after the split of the *Magnaporthaceae*. Expansion and diversification of the MAX-effectors is clearly continuing since frequently orthologs in *M*. *oryzae* or *M*. *grisea* cannot be identified and duplication, loss and diversification of MAX-effectors in host specific lineages of *M*. *oryzae* is observed (S8B Fig). Genome sequencing of additional species from *Pyricularia* and other genera in the *Periculariae* will allow to further strengthen the hypothesis of lineage-specific expansion of MAX-effectors.

Lineage specific expansion of effector families has been observed in other fungi such as mildew and rust fungi whose effector repertoires are dominated by effector families that contain frequently numerous members and are for their majority restricted to individual species or precise clades [10,51]. However, in these cases, sequence divergence is not as strong as in MAX-effectors since sequence-based comparisons allow the establishment to these effector families.

On the contrary, the effector repertoires of ascomycete phytopathogens outside the mildew lineage contain hundreds of sequence-unrelated effectors and the evolutionary origin of these huge amounts of species or clade specific genes is an open question. Duplication and diversification eventually driven by localization of the genes in transposon rich regions, genome reshuffling or transfer of accessory chromosomes were convincingly proposed as potential mechanisms to create effector diversity but the apparent lack of relatedness of ascomycete effectors remains unexplained [52–55]. Establishment of a huge effector family in *M*. *oryzae* and *M*. *grisea* that is also present at much lower frequency in other ascomycete pathogens sheds new light on the origin and relatedness of ascomycete effectors.

### Diversifying rather than convergent evolution leads to highly diversified effector families

Theoretically, convergent evolution as well as diversifying evolution can explain the situation observed for the MAX-effectors characterized by a broad and patchy distribution, high diversification and limited sequence homology as well as a shared sequence pattern and probably the same structure. Convergent evolution would apply if these proteins with similar functions and a similar fold appeared repeatedly in phytopathogenic ascomycetes and eventually evolved independently in different clades. Under diversifying evolution, a protein or protein family present in a common ancestor has been strongly diversified in different lineages of ascomycete fungi and frequently lost during evolution in certain lineages and species. The scenario of convergent evolution of MAX-effectors cannot be excluded but is clearly less parsimonious. It raises the question why MAX-effectors do not occur in organisms with similar lifestyles outside the Sordariomycete and Dothideomycete pathogens such as phytopathogenic basidiomycetes or oomycetes. In addition, there are no well-documented examples of convergent evolution towards similar folds or sequence patterns for pathogenic effectors or secreted fungal proteins involved in adaption to the environment while comparative genomics studies in fungi and oomycetes are beginning to identify such widely distributed gene families that are shaped by strong diversifying selection and that can only be properly reconstructed when pattern-based searches and structure information are taken into consideration. The best documented example is certainly the WY-domain family among the RXLR effectors that is specific to the *Peronosporales* clade in oomycetes and evolves by diversifying evolution [8,9,56–58]. Careful sequence analysis involving pattern searches identified the W, Y and L sequence motifs in the effector domains of a majority of the *Phytophtora* RXLR effectors that are frequently completely sequence unrelated [9]. Functional analysis confirmed the importance of these motifs for effector function [59] and structure analysis of the effector domain of different RXLR effectors with limited sequence homology revealed that conserved sequence motifs reflected a conserved, highly similar 3-dimensional structure named the WY-domain fold [56,60–62]. PexRD2 and AVR3a11 show e.g. only 14% amino acid identity in a structure-based alignment but overlay of their structures has an RMSD score of 0.73 Å. As in the case of the β-sandwich fold of the MAX-effectors, the WY-domain fold tolerates insertion or deletion of amino acids in the loops, exchange of surface exposed amino acids and is stabilized by hydrophobic core residues that can be exchanged as long as hydrophobicity is maintained [56]. This flexible structure allows to generate effectors with highly variable shapes and surface properties and studied WY-domain effectors showed very diverse molecular activities, target different host proteins and are recognized by different NLR immune receptors [7,56].

An example of rapidly evolving proteins from fungi that are structurally but not sequence-conserved are hydrophobins that are low molecular mass secreted proteins important for the impermeabilization of fungal cell walls, adhesion to hydrophobic surfaces and pathogenicity [63]. Hydrophobins were shown to evolve rapidly according to a birth-and-death mechanism [64], are widely distributed in a broad range of basidio- and ascomycete fungi and are characterized by sequence patterns but no sequence homology [63,65]. Structure analysis demonstrated that distantly related hydrophobins are structurally related supporting a common evolutionary origin [66].

Another example of a fungal gene family that is rapidly evolving according to a birth-and-death model are the Hce2 proteins (**h**omologs of ***C*** *ladosporium fulvum* **E**CP2) that are present in a wide range of basidio and ascomycete fungi and seem to act as effectors in pathogenic fungi and potentially in stress responses in non-pathogenic fungi [67]. Much like MAX-effectors they show patchy distribution, lineage-specific expansions and high sequence diversification.

### MAX-effectors may serve as a paradigm for the evolution and diversification of effectors in phytopathogenic ascomycetes

Based on our discovery of the MAX-effector family and the widely accepted concept that fungal effectors evolve according to a birth-and-death model we propose the hypothesis that the majority of the immense number of different ascomycete effectors could in fact belong to a restricted set of structurally defined families whose members are phylogenetically related. These families of structurally conserved effectors are expected to be, as the MAX-effectors widely distributed with frequent losses on the one hand and lineage specific expansions on the other leading to effector families that are particularly important in certain fungal clades but not in others. The evolution of individual effectors is so rapid and their adaptation to new functions so profound that sequence homology and resulting phylogenetic signals are rapidly lost although the basic protein architecture may frequently be conserved because it represents a good solution to many general constraints effectors have to face such as stability in the fungus-host interface or translocation into host cytosol. Sequence homology can therefore only be detected in orthologs from closely related species but in paralogs from the same species or homologs from more distantly related species no similarity is detected on the sequence level. Only structure-informed and pattern-based searches reveal the hidden relatedness of ascomycete effectors. This hypothesis is also supported by the recent identification of an effector super family in the powdery mildew fungus *Blumeria graminis* fsp *hordei* by structural modelling [51]. 72 effectors from different families established by sequence homology or with no homology to other proteins had 3D structure models with similarity to ribonucleases suggesting a common origin and a conserved structure in this superfamily of sequence diverse effectors.

Knowledge on the structures of fungal effector proteins is extremely limited and outside of the MAX-effectors the structures of only three cytoplasmic fungal effectors have been determined. AvrL567 from the rust fungus *Melampsora lini* and ToxA from *P*. *tritici-repentis* have distantly related β-sandwich structures whose topologies are completely different from the MAX-effectors and AvrM has a helical structure [68–70]. Therefore, the elucidation of the 3-dimensional structures of additional fungal effectors is a priority for a better understanding of their diversity and will teach us to what extent structurally conserved but sequence-diversified effector families dominate the huge and extremely diverse effector repertoires of phytopathogenic fungi.

## Methods

### Protein expression and purification

The sequence for the mature protein (residues 20–85 for AVR-Pia, and residues 23–89 for AVR1-CO39) was inserted into the pET-SP vector by ligation of PCR using NdeI-BamHI sites. PCR products were generated using the forward and reverse oligos tatcatatggctGCGCCAGCTAGATTTTGCGTCTAT and tatggatccCTAGTAAGGCTCGGCAGCAAG or tatcatatGCTTGGAAAGATTGCATCATCCA and tatggatccGATCAACAAGACTCATCGTCGTCA for respectively AVR-Pia or AVR1-CO39. The pET-SP vector was constructed from pET-15b (Merck-Millipore, Darmstadt Germany) by inserting a periplasmic secretion sequence, a hexahistidine tag and a TEV cleavage site at the N-terminus of the protein adding an extra 31 amino acid sequence at the N-terminus of the recombinant proteins (sequence MKKTAIAIAVALAGFATVAQA_APQDNTSMGSSHHHHHHSSGRENLYFQGHMA). The plasmids pET-SP-AVR-Pia and pET-SP-AVR1-CO39 were used to transform *E*. *coli* BL21 (DE3).

Transformed cells were grown in an autoinducing minimal media C-750501 [71] at 37°C for 24h. To generate isotopically-labeled samples for NMR spectroscopy, we used ^15^NH_4_Cl, ^13^C_3_-glycerol and ^13^C_6_-glucose as the primary nitrogen and carbon sources. Cells were harvested by centrifugation and the pellet was resuspended in lysis buffer (200 mM TrisHCl pH8, 200mM Sucrose, 0.05mM EDTA, 50μM lysozyme). After 30 minutes incubation, cell debris were removed by centrifugation at 12 000 *g* for 15 min at 4°C. The resulting crude protein extracts were loaded on an AKTA basic system into a HisTrap 5ml HP columm (GE Healthcare), equilibrated in buffer A (50 mM TrisHCl, pH 8.0, 300 mM NaCl, 1 mM DTT, 0.1 mM Benzamidine). The His-tagged protein was eluted from the affinity column with buffer B (buffer A supplemented with 500 mM imidazole). Fractions containing the protein were identified by SDS-PAGE and pooled. The protein was further purified by gel filtration using a Superdex S75 26/60 (GE Healthcare) column in buffer A and pure fractions were pooled.

The elution profiles indicated that AVR-Pia and AVR1-CO39 eluted as single monomeric species (Fig 1). Ellman’s reagent, 5, 5’-dithio-bis-(2-nitrobenzoic acid), DTNB, was used for quantitating free sulfhydryl groups [72]. Briefly, aliquots of standard (cysteine, Sigma, 12.5 μM to 75 μM) or sample (50 μM) were reacted with 0.1 mM DTNB reagent in 100 mM sodium phosphate pH 8.0, 1mM EDTA buffer. Free sulfhydryl groups were also measured in denaturating conditions using the same buffer supplemented with 6M Guanidinium Chloride. Absorbance was read at 412 nm on a NanoDrop 2000, and the concentration of free thiols was determined from the standard curves.

### NMR samples

The NMR samples were prepared with 1mM of purified protein at 10% D_2_O and 0.5 mM DSS as a reference. For AVR-Pia the purification buffer was exchanged with phosphate buffer (20 mM potassium-sodium phosphate, pH 5.4 and 150 mM NaCl), by filtrating with Centricon. The purified AVR1-CO39 proteins were dialyzed in 20 mM sodium phosphate, pH 6.8, 150 mM NaCl and 1 mM DTT. For the D_2_O experiments, a non-labeled sample was lyophilized and dissolved in D_2_O.

### Nuclear magnetic resonance spectroscopy

Spectra were acquired on 500 and 700 MHz Avance Bruker spectrometers equipped with triple-resonance (^1^H, ^15^N, ^13^C) z-gradient cryo-probe at 305 K. Experiments were recorded using the TOPSPIN pulse sequence library (v. 2.1) (S2 Fig). 2D-NOESY experiments with excitation sculpting water suppression were acquired at 305K, with mixing times from 100 to 150 msec. All spectra are referenced to the internal reference DSS (4,4-dimethyl-4-silapentane-1-sulfonic acid) for the ^1^H dimension and indirectly referenced for the ^15^N and ^13^C dimensions [73].

NMR data was processed using Topspin (v. 3.2) and were analyzed using strip-plots with Cindy in house software and CCPN [74] [analysis v 2.3]. Side chain assignments were carried out using 2D-NOESY, 2D-TOCSY and COSY-DQF experiments with D_2_O samples, combined with ^15^N-NOESY-HSQC and ^15^N-TOCSY-HSQC 3D spectra. For AVR-Pia, the two N-terminal residues Ala-Pro and the His-tag, Ser-His_6_-Ser were not assigned. For AVR1-CO39, the tag-residues Asp(-7)-Asn(-8) and the stretch Ser_2_-His_6_-Ser_2_ were not assigned. The ^15^N and ^13^C assignments were derived from the 3D spectra at 500 MHz.

### ^15^N backbone amide NMR relaxation data

Relaxation data were acquired at 305K on a Bruker Avance 500 MHz spectrometer using R_1_, R_2_ and ^15^N{^1^H} heteronuclear NOE pulse sequences (TOPSPIN library, v 2.1). NMR samples of 500 μL at 0.85 mM and 0.3 mM were used for AVR-Pia and AVR1-CO39, respectively. R_1_ experiments were performed with nine relaxation delays (18, 54, 102, 198, 294, 390, 582, 774 and 966 ms). R_2_ experiments were carried out employing a Carr–Purcell–Meiboom–Gill (CPMG) pulse train [75,76] with eight relaxation delays (16, 32, 48, 64, 96, 128, 192 and 256 ms). A recycle delay of 2.5 s was employed in R_1_ and R_2_, experiments, and ^15^N decoupling during acquisition was performed using a GARP-4 sequence. In heteronuclear ^15^N{^1^H}NOEs, proton saturation was achieved during the relaxation time by application of high-power 120° pulse spaced at 20 ms intervals for 3 s prior to the first pulse on ^15^N [77]. A relaxation delay equal to 6 s between each scan was used. Relaxation parameters, R_1_, R_2_ and NOEs were determined from the analysis module of CCPN [74].

### Structure calculation

The programs CYANA [78] and CNS [79] were used for automatic NOE assignments and structure calculations. The NH, Hα, ^15^N, ^13^Cα and ^13^Cβ chemical shifts were converted into Φ/Ψ dihedral angle constraints using TALOS+ (v. 1.2) [80]. The CANDID procedure of CYANA (v 2.1) was used to assign the 3D-peaks list from the ^15^N-NOESY-HSQC spectra. NOE assignments were inspected and used in a new CANDID assignment run including peaks from the 2D-NOESY spectra (with 100 and 150 msec mixing times for AVR-Pia and 100 and 200 msec for AVR1-CO39). A disulfide bridge Cys25-Cys66 for AVR-Pia and between Cys26-Cys61 for AVR1-CO39 was added based on cysteine Cβ chemical shifts and DTNB quantification of free thiols. NOE constraints were inspected and classified from very strong, strong, medium weak and very weak, corresponding to 2.4, 2.8, 3.6, 4.4 and 4.8 Å upper bound constraints, respectively. Final structure calculations were performed with CYANA (v. 2.1) using 1541 and 1286 distance restraints, for AVR-Pia and AVR1-CO39, with 90 and 72 Φ/Ψ dihedral angle constraints, respectively. The 30 conformers with lowest target function starting from 200 initial structures, were refined by CNS (v. 1.2) using the refinement in water of RECOORD [81]. The final 20 conformers were selected with the lowest NOE and dihedral angle violations. These are the structures discussed herein and deposited (PDBs, 2MYV and 2MYW). The final 20 structures contained no NOE violations greater than 0.3 Å and no dihedral angle constraint violations greater than 2°. Structures were validated using PROCHECK [82].

### Sequence analysis

Two sequence databases were used, the UniRef90 release 2015_03 [83] and a database build from the genomes of the ascomycete fungi *Magnaporthe oryzae* (reference isolate 70–15), *Colletotrichum graminicola*, *Colletotrichum higginsianum*, *Fusarium graminearum*, *Fusarium oxysporum*, *Gaeumannomyces graminis*, *Magnaporthe poae*, *Neurospora crassa*, *Pyrenophora tritici-repentis*, *Verticillium dahliae*, *Aspergillus fumigatus*, *Aspergillus nidulans*, *Blumeria graminis*, *Botrytis cinerea*, *Colletotrichum gloeosporioides*, *Colletotrichum orbiculare*, *Dothistroma septosporum*, *Fusarium fujikuroi*, *Fusarium pseudograminearum*, *Fusarium verticillioides*, *Leptosphaeria maculans*, *Phaeosphaeria nodorum*, *Pyrenophora teres*, *Trichoderma virens*, *Tuber melanosporum* and *Zymoseptoria tritici* (all from the Ensembl Fungi database http://fungi.ensembl.org) as well as the genomes of eight *M*. *oryzae* isolates specific for *Eleusine coracana* (CD156), *Triticum aestivum* (BR32), *Setaria italica* (US71) and *Oryza sativa* (TH16, GY11, FR13, TH12, PH14) and one *M*. *grisea* isolate (BR29) pathogenic to *Digitaria* ssp (genome sequences at http://genome.jouy.inra.fr/gemo) [47]. Sequences without signal peptide (according to SIGNALP 4.1 [84]) bigger than 170 amino acids or with less than 2 cysteine residues were removed. For the initial HMM search, identical sequences were reduced to only one occurrence in the databases.

The start of the search was a structural alignment with TM-align [85] and the structures of AVR-Pia, AVR1-CO39, AvrPiz-t and ToxB complemented with sequence homologues found by single queries using BLAST (v 2.2.27+) with a stringent cut-off E-value = 1e-6. For the ToxB query, two iterations of NCBI PSI-BLAST were used on the NR database with a cut-off E-value = 1e-4 (S5A Fig).

This initial alignment was used as input to look for homologs in the filtered and non-redundant fungi database using HMMERsearch program from the HMMER package v 3.0 [86] with a 1e-6 E-value cut-off. For each run, only sequences where the two cysteine residues were aligned were kept, and the output alignment was used as input query for a new HMMERsearch run. This run was repeated until reaching convergence. New iterations were then done with increased E-value cut-off at 1e-5 and 1e-4. From the last alignment, a histogram indicated that the two aligned cysteine residues were separated by at least 34 and at most 49 amino acids.

The full homolog search was re-started, as described above, but this time using also the aligned cysteine separations as an additional constraint for filtering homologs after each HMMERsearch run. The HMMERsearch runs were repeated until convergence for raised threshold E-values 1e-6, 1e-5, 1e-4 and finally 1e-2. The homolog ensembles obtained for the three E-values cut-off, 1e-6, 1e-4 and 1e-2 were aligned with Muscle v3.8 [87] (S6A Fig for E-value 1e-4). The derived logo was built from the HMMER search with E-value of 1e-4 using Weblogo3 [88]. The multiple sequence alignment (MSA) derived from the HMMER search with E-value 1e-4 was used as input to look for homologs in the redundant fungi database and the UniRef90 database, using HMMERsearch with an E-value threshold of 1e-1. Diversity trees were built from alignments generated with Muscle v3.8 using the Neighbor-Joining method with the MEGA6 package [89].

### Fungal growth and infection assays

For analysis of gene expression *in vitro* grown mycelium, *M*. *oryzae* isolate Guy11 was grown in liquid medium (glucose 10g/L, KNO_3_ 3g/L, KH_2_PO_4_ 2g/L and yeast extract 2g/L) at 120 rpm on a rotary shaker at 25°C for five days. Mycelium was harvested over a piece of cheese-cloth (Merck-Millipore, Darmstadt Germany).

For production of spores for infection assays, *M*. *oryzae* isolate Guy11 was grown on rice flour agar for spore production [90]. A suspension of fungal conidiospores was prepared at a density of 2x10^5^ spores/ml and spotted on detached leaves of the japonica rice variety Saraceltik grown for 3 weeks as described [91]. Infected leaf samples were harvested 16, 24, 48 and 72 hours post inoculation (hpi).

### RNA extraction and qRT–PCR analysis

RNA extraction and reverse transcription was performed as described [92]. Quantitative PCR were performed with a LightCycler 480 instrument (Roche, Basel, Switzerland) using LC 480 SYBR Green I Master Mix (Roche) and the primers listed in the S4 Table. Amplification was performed as follows: 95°C for 10 min; 40 cycles of 95°C for 15 s, 60°C for 20s and 72°C for 30 s; then 95°C for 5 min and 40°C for 30 s. Data were analyzed using the delta-delta Ct method and applying the formula 2-∆CT, where ∆CT is the difference in threshold cycle (CT) between the gene of interest and the housekeeping gene *Actin* (*MGG_03982*) used as a constitutively expressed reference gene. For each condition, three biological replicates were analyzed.

## Supporting Information

S1 Table NMR experiments acquired for structure calculations and chemical shift assignments. (PDF)

S2 Table DALI statistics for structural alignment of AVR-Pia, AVR1-C039, AvrPiz-t and ToxB. (PDF)

S3 Table MAX-effector candidates identified by psi-Blast in the genomes of the *M*. *oyzae* isolates 70–15 and TH16 and the *M*. *grisea* isolate BR29. (PDF)

S4 Table Primers used for qRT-PCR. (PDF)

S1 Fig Gel filtration profile and SDS-PAGE analysis of purified AVR-Pia (A) and AVR1-CO39 (B) proteins. (PDF)

S2 Fig 15N Relaxation data at 500 MHz and 305K for AVR-Pia (panels A, B and C) and AVR1-CO39 (panels D, E and F). (PDF)

S3 Fig Backbone sequential and medium range NOEs observed for (A) AVR-Pia and (B) AVR1-CO39. The line width is proportional to the NOE intensity. The dots (•) indicate slow exchange NH observed in 2D-NOESY in D2O. Grey arrows indicate the ß-strands determined from the structure analysis (vide infra).
(PDF)

S4 Fig Solution structures of (A) AVR-Pia and (B) AVR1-CO39. Superposition of the backbone atoms of the 20 lowest energy conformers used to calculate the final structures. Only mature chains are shown, from residues Ala20 and Trp23 for AVR-Pia and AVR1-CO39, respectively.
(PDF)

S5 Fig Structure-guided alignment and diversity of MAX-effector homologs identified by Blast. A) Homologs of AVR1-CO39, AvrPiz-t and AVR-Pia identified by Blast in *M*. *oryzae* and *M*. *grisea* genomes and ToxB homologs identified by Psi-Blast in the GeneBank database were aligned to the structural alignment of mature ToxB, AVR1-CO39, AvrPiz-t and AVR-Pia. (B) A diversity tree was constructed by the neighbor-joining method using the alignment in (A). It highlights the high diversity of MAX-effector homologs. Branch supports are based on 1000 bootstraps and horizontal branch length reflects sequence divergence. Accession numbers of non-Magnaporthe sequences were completed by a 2 letter identifier for the species: BO for *Bipolaris oryzae*, CF is for *Colletotrichum fioriniae*, CH for *C*. *higgensianum*, CG for *C*. *gloeosporioides*, CO for *C*. *orbiculare*, LM for *Lepthosphaeria maculans*, PT for *Pyrenophora tritici-repentis* and PB for *Pyrenophora bromi*.
(PDF)

S6 Fig MAX-effector homologs identified by a high stringency HMM search. (A) Histogram showing the numbers of MAX-effectors identified by an HMM pattern search in a non-redundant database comprising the small secreted proteins of 25 ascomycete fungi and of 8 additional *M*. *oryzae* and one *M*. *gisea* isolate. (B) MAX-effectors were aligned to the structural alignment of mature ToxB, AVR1-CO39, AvrPiz-t and AVR-Pia and gaps were removed. (C) A diversity tree was constructed by the neighbor-joining method using the alignment in (B). Branch supports are based on 1000 bootstraps and horizontal branch length reflects sequence divergence. Accession numbers of non-Magnaporthe sequences were completed by a 2 letter identifier for the species: BO for *Bipolaris oryzae*, CF for *Colletotrichum fioriniae*, CH for *C*. *higgensianum*, CG for *C*. *gloeosporioides*, CO for *C*. *orbiculare*, LM for *Lepthosphaeria maculans*, PT for *Pyrenophora tritici-repentis* PB for *Pyrenophora bromi* and ZT for *Zymoseptoria tritici*.
(PDF)

S7 Fig MAX-effector homologs identified in the UniRef90 database by a low stringency HMM search. (A) Histogram showing the numbers of MAX-effectors identified by an HMM pattern search in a non-redundant UniRef90 database. (B) MAX-effectors identified by HMM pattern search were aligned to the structural alignment of mature ToxB, AVR1-CO39, AvrPiz-t and AVR-Pia. (C) A diversity tree was constructed by the neighbor-joining method using the alignment in (B). This highlights the high diversity of MAX-effector homologs. Branch supports are based on 1000 bootstraps and horizontal branch length reflects sequence divergence. Accession numbers contain the following information on the species: MAGGR, MAGO7, MAGOP and MAGOR are from *M*. *oryzae*, COLGC and COLGN from *C*. *gloeosporioides*, COLHI from *Colletotrichum higginsianum*, 9PEZI from *C*. *fioriniae* and COLOR *from Colletotrichum orbiculare*, 9PLEO from*P*. *tritici-repentis* or *P*. *bromi*, ARTOA from *Arthrobotrys oligospora*, COCMI from *Bipolaris oryzae*, LEPMJ from *Leptosphaeria maculans*, MYCGM from *Zymoseptoria tritici*, 9PSED from *Pseudomonas sp*. *StFLB209* and SOLLC from *Solanum lycopersicum*.
(PDF)

S8 Fig MAX-effector homologs identified by a low stringency HMM search in the fungal genomes database. (A) MAX-effectors identified by an HMM pattern search in a redundant database comprising the small secreted proteins of 25 ascomycete fungi, 8 additional *M*. *oryzae* and one *M*. *gisea* isolate were aligned to the structural alignment of mature ToxB, AVR1-CO39, AvrPiz-t and AVR-Pia and gaps were removed. (B) A diversity tree was constructed by the neighbor-joining method using the alignment in (A). Branch supports are based on 1000 bootstraps and horizontal branch length reflects sequence divergence. Accession numbers of non-Magnaporthe sequences were completed by a 2 letter identifier for the species: BO for *Bipolaris oryzae*, CF for *Colletotrichum fioriniae*, CH for *C*. *higgensianum*, CG for *C*. *gloeosporioides*, CO for *C*. *orbiculare*, GF for *Fusarium fujcuroi*, LM for *Lepthosphaeria maculans*, PT for *Pyrenophora tritici-repentis*, PB for *Pyrenophora bromi* and ZT for *Zymoseptoria tritici*.
(PDF)

S9 Fig Expression of *M*. *oryzae* MAX-effector candidates and marker genes during rice infection and in *in vitro* grown mycelium. mRNA levels of *M*. *oryzae* genes coding for MAX-effectors (A, B and C) and marker genes (D) was determined by q-RT-PCR in rice leaf samples harvested 16, 24, 48 or 72 h after inoculation and mycelium grown liquid medium for 72 hours. (A) Infection specific MAX-effectors identified in the HMM search, (B) infection specific MAX-effectors identified in the Psi Blast search but nor in the HMM search, (C) constitutively expressed MAX-effectors identified in the HMM search and (D) marker genes for appressorium and very early infection (*ORF3* of the ACE1 cluster, *MGG_0838*1), biotrophic infection (*BAS3*, *MGG_11610*), late infection (*MGG_01147*), constitutive expression (*EF1α*, *MGG_03641*). Relative expression levels were calculated by using expression of a constitutively expressed *Actin* (*MGG_03982*) as a reference. Mean values and standard deviation were calculated from three independent biological samples.The analyzed genes, were in most cases not or extremely weakly expressed in the mycelium. For genes with significant expression in the mycelium (ratio gene versus actine > 0,01) a T-test was performed to determine if in planta expression was significantly different from expression in the mycelium. In these cases (MGG_11967, MGG_14793, MGG_15207, MGG_17266, MGG_18019, M_TH16_00000541, M_TH16_00040131, M_TH16_00079081, M_TH16_00104561, M_TH16_00120731, M_TH16_00124981), a star or two stars (* or **) mark conditions where the expression was different from expression in the mycelium at respectively p<0,05 or p<0,005.
(PDF)

S10 Fig Prediction of the secondary structure of M. oryzae MAX effectors. The secondary structures of the MAX-effectors from the 70–15, TH16 and BR29 genomes was predicted with SSPRO5 [93].The predictions are shown at the bottom of the figure and are aligned onto the corresponding primary sequence alignment shown at the top of the figure. Sequence identifiers for the secondary structure predictions are suffixed with ".2d.SSPRO5". Blue"H", red "E" and yellow "C" correspond respectively to helix, extended sheet and coil predictions. The sequences of the 4 MAX effectors with experimentally determined structures are displayed at the top of the multiple sequence alignment and, for clarity, the alignment positions corresponding to shared gaps in the known structures were removed.
(TIF)
