# Supplementary material for: Structure Analysis Uncovers a Highly Diverse but Structurally Conserved Effector Family in Phytopathogenic Fungi
Source: PLoS Pathog. 2015 Oct 27;11(10):e1005228. doi: 10.1371/journal.ppat.1005228 (PMC4624222; doi:10.1371/journal.ppat.1005228)
Supplement: S1 Table — (PDF) [file ppat.1005228.s001.pdf]

Supplemental Table S1. *NMR experiments acquired for structure calculations and chemical shift assignments*

a) AVR-Pia

		Size			Sweep width (ppm)						
Experiments	nuclei	F3	F2	F1	F3	F2	F1	Mix(ms)	NS	D1(s)	B_o(MHz)
^{15}N-HSQC	^{1}H, ^{15}N	1024	128	-	-	-	-	-	16	1	700
HNCO(*)	^{1}H,^{15}N,^{13}C	1024	64	64	12.98	36	17.67	-	8	0.001	500
HNCA(*)	^{1}H,^{15}N,^{13}C	1024	64	80	12.98	36	31.81	-	16	0.1	500
HNCOCACB(*)	^{1}H,^{15}N,^{13}C	1024	64	128	12.98	36	75.02	-	32	0.2	500
HNCACO(*)	^{1}H,^{15}N,^{13}C	1024	64	80	12.98	36	17.67	-	64	0.001	500
HNCACB(*)	^{1}H,^{15}N,^{13}C	1024	64	128	12.98	36	75.02	-	32	0.2	500
CON(**)	^{13}C, ^{15}N	1024	200	-	30	36	-	-	4	1	500
CACO(**)	^{13}C, ^{13}C	1024	256	-	30	50.32	-	-	8	1	500
^{15}N-NOESY-HSQC	^{1}H,^{15}N, ^{1}H	1500	76	360	14	35	14	120	8	1	700
^{15}N-TOCSY-HSQC	^{1}H,^{15}N, ^{1}H	1500	70	320	14	35	14	56	8	1	700
NOESY (D_2O)	^{1}H, ^{1}H	2048<br>4096	512<br>512	-	15.00<br>12.01	15.00<br>12.01	-<br>-	100<br>150	128<br>128	1<br>1	700<br>700
TOCSY (D_2O)	^{1}H, ^{1}H	4096	512	-	12.01	12.01	-	39.6	64	1	700
COSY-dqf (D_2O)	^{1}H, ^{1}H	4096	800		12.01	12.01	-	-	64	1	700
^{1}H,^{15}N NOE	^{1}H, ^{15}N	1024	128	-	14	35	-	Sat. 3s	64	6	500
R_1	^{1}H, ^{15}N	1024	128	-	14	35	-		16	2.5	500
R_2	^{1}H, ^{15}N	1024	128	-	14	35	-		16	2.5	500

b) AVR1-CO39

		Size			Sweep width (ppm)						
Experiments	nuclei	F3	F2	F1	F3	F2	F1	Mix(ms)	NS	D1(s)	B_o(MHz)
^{15}N-HSQC	^{1}H, ^{15}N	1500	160	-	12.02	28	-	-	16	1	700
1^{3}C-HSQC	^{1}H, ^{13}C	2048	256	-	15.00	80	-	-	512	1.5	700
HNCO(*)	^{1}H,^{15}N,^{13}C	1024	64	64	12.02	28	17.67	-	8	0.001	500
HNCA(*)	^{1}H,^{15}N,^{13}C	1024	64	80	18.02	28	31.81	-	16	0.1	500
HNCOCACB(*)	^{1}H,^{15}N,^{13}C	1024	64	128	18.02	28	75.02	-	32	0.2	500
HNCACO(*)	^{1}H,^{15}N,^{13}C	1024	64	80	18.02	28	17.67	-	64	0.001	500
HNCACB(*)	^{1}H,^{15}N,^{13}C	1024	64	128	18.02	28	75.02	-	32	0.2	500
CON(**)	^{13}C, ^{15}N	1024	200	-	30	28	-	-	4	1	500
CACO(**)	^{13}C, ^{13}C	1024	256	-	30	50.32	-	-	2	1	500
^{15}N-NOESY-HSQC	^{1}H,^{15}N, ^{1}H	1500	76	360	12	28	12	120	8	1	700
^{15}N-TOCSY-HSQC	^{1}H,^{15}N, ^{1}H	1024	72	320	14	35	14	56	8	1	700
NOESY (D_2O)	^{1}H, ^{1}H	2048<br>2048	512<br>512	-	15.00<br>15.00	15.00<br>15.00	-<br>-	100<br>200	64<br>32	1<br>1	700<br>700
TOCSY	^{1}H, ^{1}H	2048	512	-	15.00	15.00	-	40	32	1	700
COSY-dqf	^{1}H, ^{1}H	2048	800		15.00	15.00	-	-	32	1	700
^{1}H,^{15}N NOE	^{1}H, ^{15}N	1024	128	-	14	35	-	Sat. 3s	64	6	500
R_1	^{1}H, ^{15}N	1024	128	-	14	35	-		16	2.5	500
R_2	^{1}H, ^{15}N	1024	128	-	14	35	-		16	2.5	500

Experiments were recorded using the TOPSPIN Library (v. 2.1) at 305 K.

(*) Best pulse sequences

(**)IPAP scheme for virtual decoupling
