# Supplementary material for: Structure Analysis Uncovers a Highly Diverse but Structurally Conserved Effector Family in Phytopathogenic Fungi
Source: PLoS Pathog. 2015 Oct 27;11(10):e1005228. doi: 10.1371/journal.ppat.1005228 (PMC4624222; doi:10.1371/journal.ppat.1005228)
Supplement: S2 Table — (PDF) [file ppat.1005228.s002.pdf]

S2 Table. DALI Statistics for structural alignment of AVR-Pia, AVR1-CO39, AVRPiz-t and ToxB.

**RMSD (Å)** / **Z-score**	AVR-Pia	AVR1-CO39	AVRPiz-t	ToxB
AVR-Pia		2.3	2.8	2.2
AVR1-CO39	2.9		3.0	2.2
AVRPiz-t	3.3	3.1		2.2
ToxB	4.4	4.2	5.4	

The rmsd (upper right) and the Z-score (lower left) are from the pairwise superposition. The low RMSDs and the high Z-scores indicate that the ToxB(b) structure is the closest to the other three structures.
