# Supplementary material for: Structure Analysis Uncovers a Highly Diverse but Structurally Conserved Effector Family in Phytopathogenic Fungi
Source: PLoS Pathog. 2015 Oct 27;11(10):e1005228. doi: 10.1371/journal.ppat.1005228 (PMC4624222; doi:10.1371/journal.ppat.1005228)
Supplement: S3 Table — (PDF) [file ppat.1005228.s003.pdf]

S3 Table MAX-effector candidates identified by psi-Blast in the genomes of the *M. oyzae* isolates 7015 and TH16 and the *M. grisea* isolate BR29.

geneID	localization	AVR-Pia*	AVR1-CO39*	ToxB*
M_BR29_EuGene_00004921	scaffold00001	9,0E-12	-	6,0E-12
M_BR29_EuGene_00041131	scaffold00007	-	9,0E-06	-
M_BR29_EuGene_00043011	scaffold00008	-	9,0E-06	-
M_BR29_EuGene_00060181	scaffold00013	-	4,0E-08	-
M_BR29_EuGene_00081821	scaffold00023	-	3,0E-07	-
M_BR29_EuGene_00082031	scaffold00023	-	1,0E-05	-
M_BR29_EuGene_00085071	scaffold00025	-	-	3,0E-15
M_BR29_EuGene_00087671	scaffold00027	4,0E-13	4,0E-12	8,0E-11
M_BR29_EuGene_00088411	scaffold00027	-	-	5,0E-12
M_BR29_EuGene_00091361	scaffold00030	3,0E-12	-	-
M_BR29_EuGene_00091681	scaffold00031	-	2,0E-05	-
M_BR29_EuGene_00095641	scaffold00035	-	3,0E-13	5,0E-13
M_BR29_EuGene_00106461	scaffold00049	7,0E-23	-	-
M_BR29_EuGene_00107481	scaffold00051	-	2,0E-06	-
M_BR29_EuGene_00112111	scaffold00059	-	6,0E-09	-
M_BR29_EuGene_00113041	scaffold00061	-	7,0E-08	-
M_BR29_EuGene_00118801	scaffold00076	-	2,0E-05	-
M_BR29_EuGene_00119491	scaffold00079	-	3,0E-22	-
M_BR29_EuGene_00119511	scaffold00079	-	9,0E-17	-
M_BR29_EuGene_00121691	scaffold00087	-	1,0E-13	-
M_BR29_EuGene_00125811	scaffold00145	-	7,0E-08	-
M_BR29_EuGene_00126081	scaffold00163	-	-	2,0E-20
MGG_00821	Chromosome_8.5	-	6,0E-14	-
MGG_04384	Chromosome_8.2	-	2,0E-04	-
MGG_08482	Chromosome_8.4	-	1,0E-06	-
MGG_08944	Chromosome_8.2	-	-	5,0E-09
MGG_10120	Chromosome_8.4	-	5,0E-04	-
MGG_14793	Chromosome_8.2	4,0E-19	3,0E-06	9,0E-15
MGG_14834	Chromosome_8.4	-	2,0E-05	-
MGG_15207	Chromosome_8.3	-	5,0E-08	-
MGG_15459	Chromosome_8.1	-	8,0E-08	-
MGG_16058	Chromosome_8.1	-	1,0E-05	-
MGG_16113	Chromosome_8.1	-	3,0E-09	-
MGG_16175	Chromosome_8.1	-	5,0E-08	-
MGG_16619	Chromosome_8.3	-	3,0E-06	-
MGG_17132	Chromosome_8.4	2,0E-06	7,0E-04	9,0E-10
MGG_17255	Chromosome_8.4	-	1,0E-06	-
MGG_18019	Chromosome_8.7	-	7,0E-05	8,0E-11
MGG_18060	Chromosome_8.7	5,0E-14	5,0E-14	4,0E-06
M_TH16_EuGene_00000541	scaffold00001	-	1,0E-14	-
M_TH16_EuGene_00027191	scaffold00004	4,0E-10	-	-
M_TH16_EuGene_00027411	scaffold00004	4,0E-12	-	-
M_TH16_EuGene_00034081	scaffold00004	-	3,0E-08	-
M_TH16_EuGene_00040131	scaffold00005	2,0E-25	-	-
M_TH16_EuGene_00045871	scaffold00007	-	3,0E-12	-
M_TH16_EuGene_00079081	scaffold00016	6,0E-10	-	-
M_TH16_EuGene_00079311	scaffold00016	-	2,0E-08	-
M_TH16_EuGene_00099371	scaffold00026	-	3,0E-08	-
M_TH16_EuGene_00101881	scaffold00028	-	2,0E-05	2,0E-08
M_TH16_EuGene_00106621	scaffold00033	-	-	5,0E-14
M_TH16_EuGene_00120731	scaffold00052	3,0E-06	-	-
M_TH16_EuGene_00124981	scaffold00063	-	7,0E-11	-
M_TH16_EuGene_00127871	scaffold00072	-	2,0E-21	-
M_TH16_EuGene_00134971	scaffold00110	-	9,0E-14	1,0E-14
M_TH16_EuGene_00135161	scaffold00112	-	-	2,0E-19

Orthologs of 7015 proteins in TH16 are not listed.

* Values correpond to e-values in psi-blast search with the corresponding protein
