# Supplementary material for: Structure Analysis Uncovers a Highly Diverse but Structurally Conserved Effector Family in Phytopathogenic Fungi
Source: PLoS Pathog. 2015 Oct 27;11(10):e1005228. doi: 10.1371/journal.ppat.1005228 (PMC4624222; doi:10.1371/journal.ppat.1005228)
Supplement: S4 Table — (PDF) [file ppat.1005228.s004.pdf]

S 4 Table Primers used for qRT-PCR

GENE	Forward	Reverse
**Infection specific expression**		
**MGG_00954**	GTACCCAAGCCCTGGAAAGT	CCAGGACAATGCTCAAATCA
**MGG_02207**	GTGCCAATTGGTGGCTTACT	TCTTTCGCTTAGCGTCGTAAC
**MGG_02546**	AAGCTACGCCGTTTGTGAAC	CGGGTAATTTCTCGCTGTCA
**MGG_04735**	GCAAAGTCGTCACCGAGATT	CGTCCATTGTCCCTTCAATTA
**MGG_05896**	GACGTTTGCCTGGGTTAAAG	GGCAAGTGTAGCCGTTGATAA
**MGG_08414**	CGTCGTGGGTGATTTGAAC	GTACCAATTCCACGCCAGTC
**MGG_08482**	ACCATGCAAGGTCACGATTT	TGATATGTGTGGCCGGTAGA
**MGG_08944**	CCTGACCAATGGCAACAAG	AAATTCGCACTTGGAGTTCA
**MGG_08992**	AAGAGACGGGAACGTTGATG	ATCCACGTCGTACCGCTTAT
**MGG_09675**	CCGTCAAAGAGGAAGGATTC	TTTGGCAGAATGCAATTCAC
MGG_11967	ATGGGCTCAAAGCTCTTGTC	TGGATTTCTTGGTCCCAGTT
MGG_13133	AGTCGAAAGGGTTGGGAGAT	ATCGGATTGCACTCCTTGTC
**MGG_15207**	CCCACCTCAATCCTGAGAGA	TATCTCGCTCCCACGGTTAC
**MGG_15625**	CAGCAAAGAAGGTGGGAAGA	GCCGATACACATTTGCACTG
**MGG_15972**	TCGAGAGCGAGACCCTAACT	CCCAGTTTGGACCAACTTTC
**MGG_16175**	CGATTGCACACTTGGTTGTAA	TGCATCCTCTTCCGATCTTT
**MGG_16619**	GAAGCTAGGCGATCGTTCTG	GCAATCCTGTTCCACATGAA
**MGG_16939**	ATTGCTGGCCAAGGTTGAG	CGGCAGTTTCTGAACATGAG
**MGG_17266**	AAACACCAGCTCCCATCAAC	GCTGGAACTAGGCGACTCTG
**MGG_18019**	TGCCTTCCAGCCTACAGAGT	CTTTCGGAACGCTCTTCTTG
**MGG_18062**	CAACAGCAGCTTCGATATCAT	CCAACTTTGGCAAGCAATTC
**M_TH16_00000541**	GGAAATCAGCGGTTTCAAGT	CAAACTTCCAGCTCGTGACA
**M_TH16_00027411**	AGAGTCGAAGCAGGCGAAAT	TTCGCGGAGACTGTACATGA
**M_TH16_00034081**	AATCAGACGCCTGAGCAAGT	GCGATCGCAGGTAATGTAGC
**M_TH16_00040131**	GCTTGGCTGGGTTAAGATTG	TTTGCAGTTGGTGTCCTCTG
**M_TH16_00079081**	GGTTGGTGCAAGGTCAAAGT	CCGTCGAGATTCCAATGAGT
**M_TH16_00079311**	AAAGCTGCATGGCAAGACT	AACCTCCGATTGTTCCACTG
**M_TH16_00104561**	CGTCGTCGATTCAGCAAATA	GCTGCTTCCTTATCCCACAA
**M_TH16_00119711**	CAGCGGTGTAGTGGCAATAA	TCCTTCCCAGCTTTCACAGT
**M_TH16_00120731**	ACCGTGGGAATGGGTTACTT	TAAACTCCCGGCTGGATAAG
**M_TH16_00124981**	GCTTGGGTTGCACCTACAGT	TTCAAACTCGCAGGTGTCTG
**M_TH16_00127871**	GTTGCCTGTGCAGCCTATCT	GATTCGGCTGGAATCATCTC
**M_TH16_00136331**	AAGGAAGAAGTCGAGGGTGAG	AACGATATCCCAACCGTCAG
**Constitutive expression and controls**		
**MGG_14793**	ACAGCCGTCTGCGACTTTAT	GTCCGCTCAGGCTAAGTTTG
**MGG_07184**	AGCGTGTCTGCAAAGCTGTA	AGACCTCCCAACGGTTCTCT
**Actin, MGG_03982**	TCTTCGAGACCTTCAACGCC	ACCGGAGTCGAGCACGATAC
**EF1α , MGG_03641**	GCC CGG TAT GGT CGT TAC CT	AGC TGC TGG TGG TGC ATC TC
**ORF3, MGG_08381**	GGTGAGGGTGTTGGAGGTAGTG	TGGAGCTGCCCAACATGTC
**MGG_01147**	CGACGACCTACCTGCTGACT	CAATGCTCCTCTTCCTGGAG
**BAS3, MGG_11610**	CCCGTGTTTGAGGAATTGTG	CTTGAGGTTGTCGGTGCTCT
**No expression**		
**MGG_00821**	GCGGCTACACGATTGAGAC	**ACCTCGTGGCTATTCCTGAC**
**MGG_08469**	CCAGACGTACCTCCCACTTG	**AGGCCCTCCAATTTCTTCTC**
**MGG_08607**	ACGCAACAACCGAAGAAATC	**GGTCCAACCGGAACTGATAA**
**MGG_09425**	TAGCCAGGAAGGCACTTACA	**GCGATCAAATCGAACCTTGA**
**MGG_10335**	ATCCTTGTAGGCGCAAAGTG	**GCAGGTTAATGCCTTTGACA**
**MGG_12426**	GGCAGGAGAGTCACCTTCAG	**CGGAGATGATGCAACGTTTA**
**MGG_15106**	TTTGGATCACCGGGAAATAC	**CTCGCCGCCAATAAACTTTC**
**MGG_15575**	AGGACTTGCCAAAGGGATTT	**GCGCCAAGAACTCATCAATC**
**MGG_16113**	GGGTTATGGCACGGTTGTTA	**GCCTGAGGCAGTTTCAGTTC**
**MGG_16416**	GCAACTCATTCGCCACAGTA	**GCAATGCCCATAACCAGTCT**
**MGG_17132**	AAATGCAATTCAAATCCCTCTT	**ACCATCTCCTCCGGATTTCT**
**MGG_17255**	AGTCGTGTCACTGGCATCAG	**GCCACCAATTGACGTTCTTT**
**MGG_17799**	TTGGCACGAAATGTGCTATT	**TCGTAGTCCAACCGGACTGT**
**MGG_18041**	GGGTTGGGCCGTTAGATTT	**CCAAATTGGTGCATTTCAGA**
**MGG_18060**	GGGTGTCCATATTCCGTGAG	**TGATCGGCTGAGATTGTAGC**
**M_TH16_EuGene_00099371**	TCCTGGCTTCGTTCAATAAGA	**TCGAATGATAGTGCGGGTAAC**
**M_TH16_EuGene_00101881**	GTGCAAGGTCGAGATGTTTG	**CGGACCAGTTCTTGGGTAAG**
**M_TH16_EuGene_00106621**	TGCAAATTCAACGTCCTCAA	**GTGAAAGGGCTGACGGTAAC**
