# Supplementary figures and images for: Structure Analysis Uncovers a Highly Diverse but Structurally Conserved Effector Family in Phytopathogenic Fungi
Source: PLoS Pathog. 2015 Oct 27;11(10):e1005228. doi: 10.1371/journal.ppat.1005228 (PMC4624222; doi:10.1371/journal.ppat.1005228)

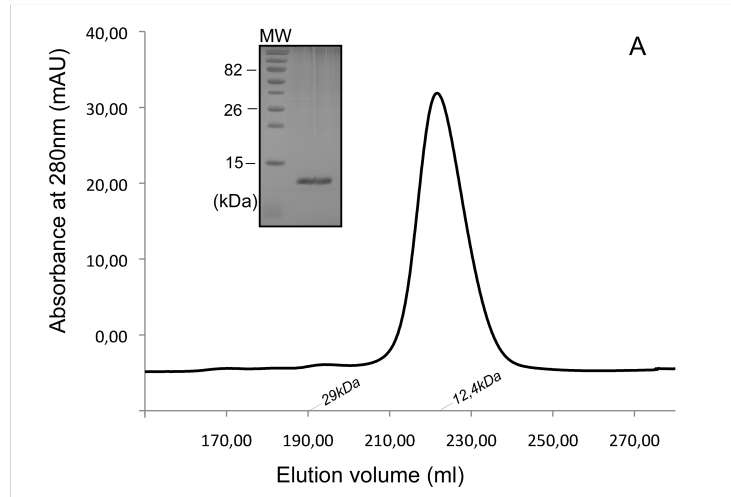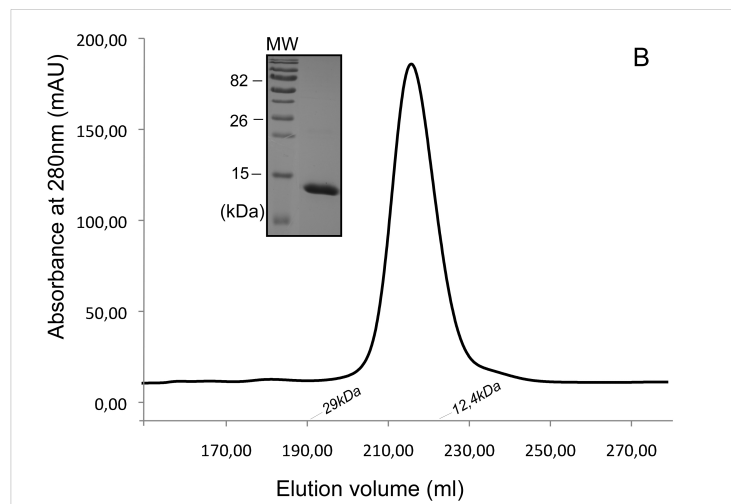

Supplement: S1 Fig — (PDF) [file ppat.1005228.s005.pdf]

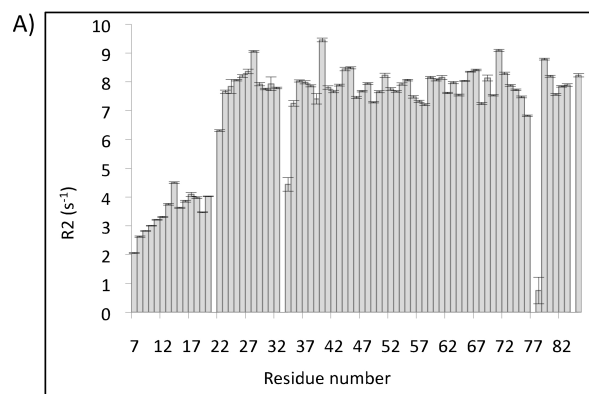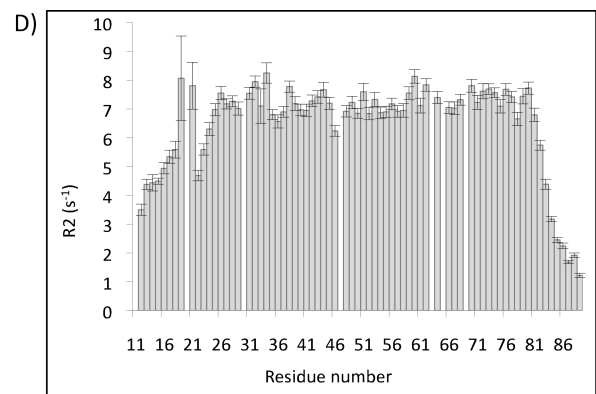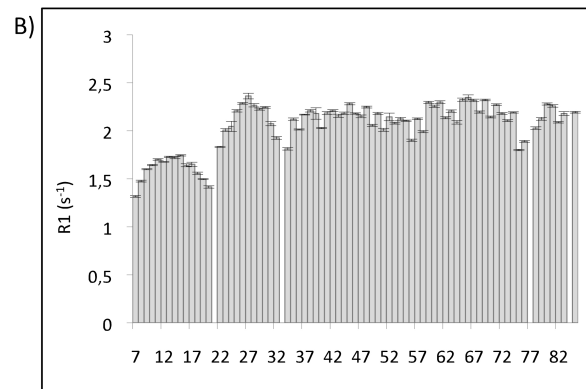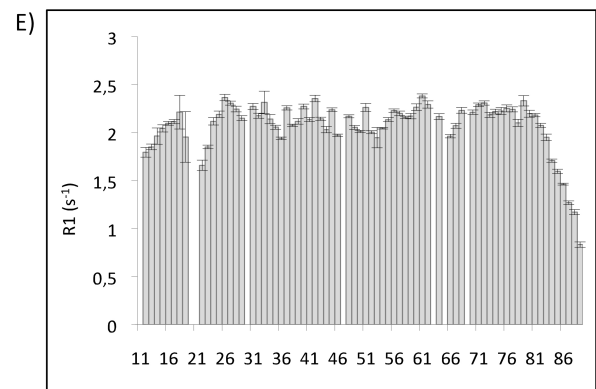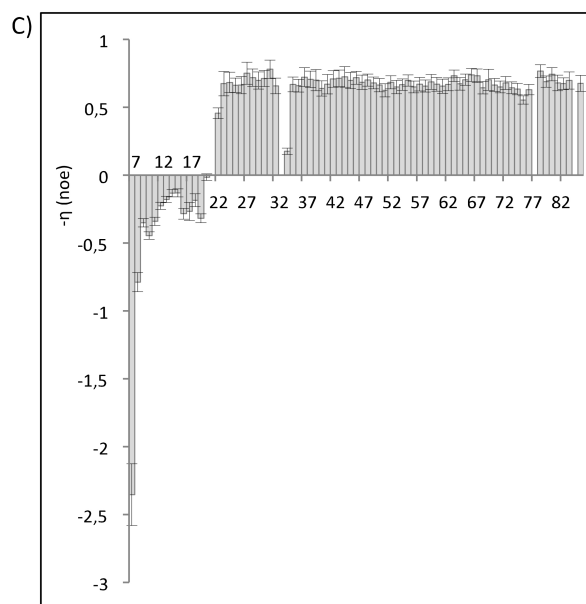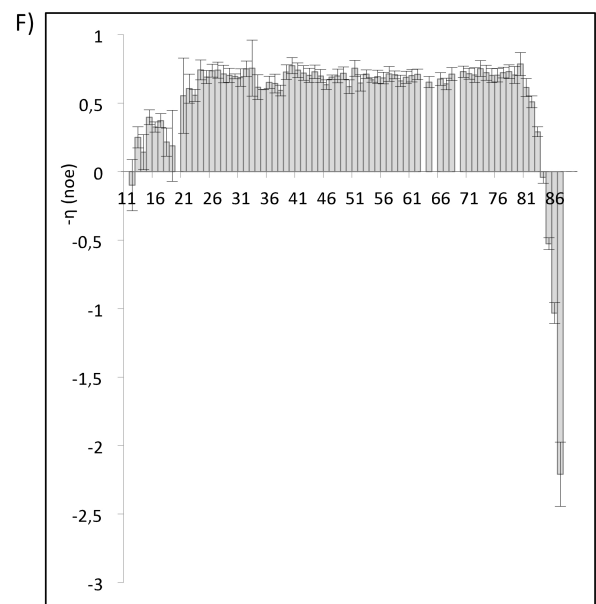

Supplement: S2 Fig — (PDF) [file ppat.1005228.s006.pdf]

(A) AVR-Pia

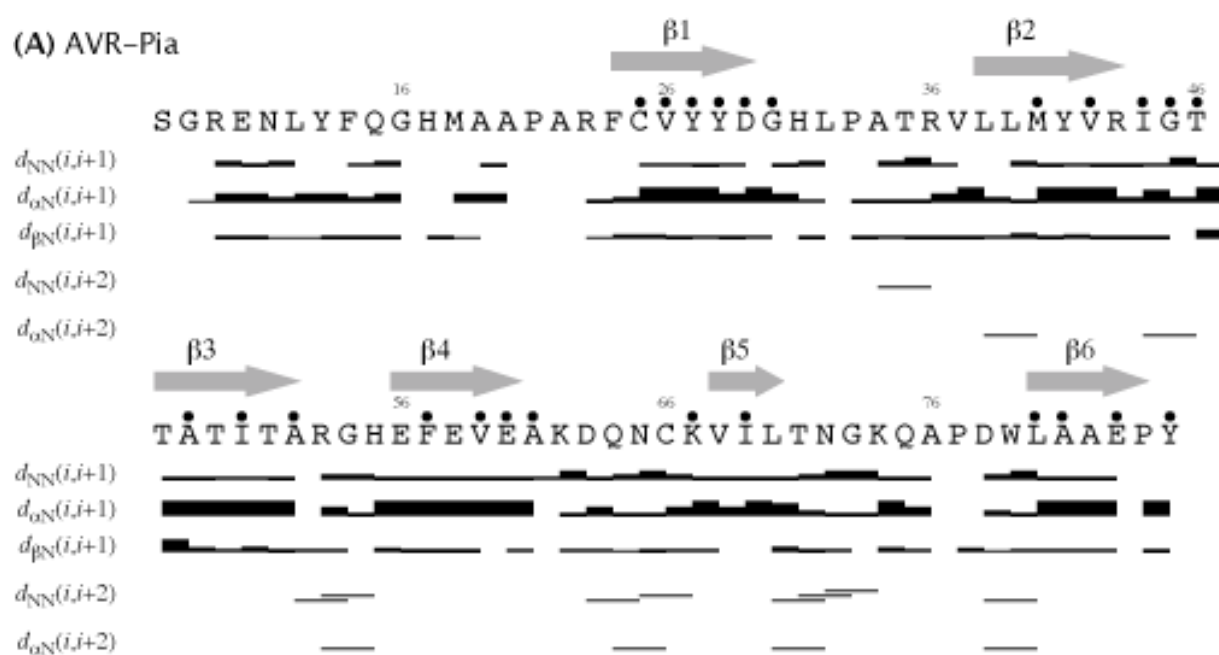

(B) AVR1-CO39

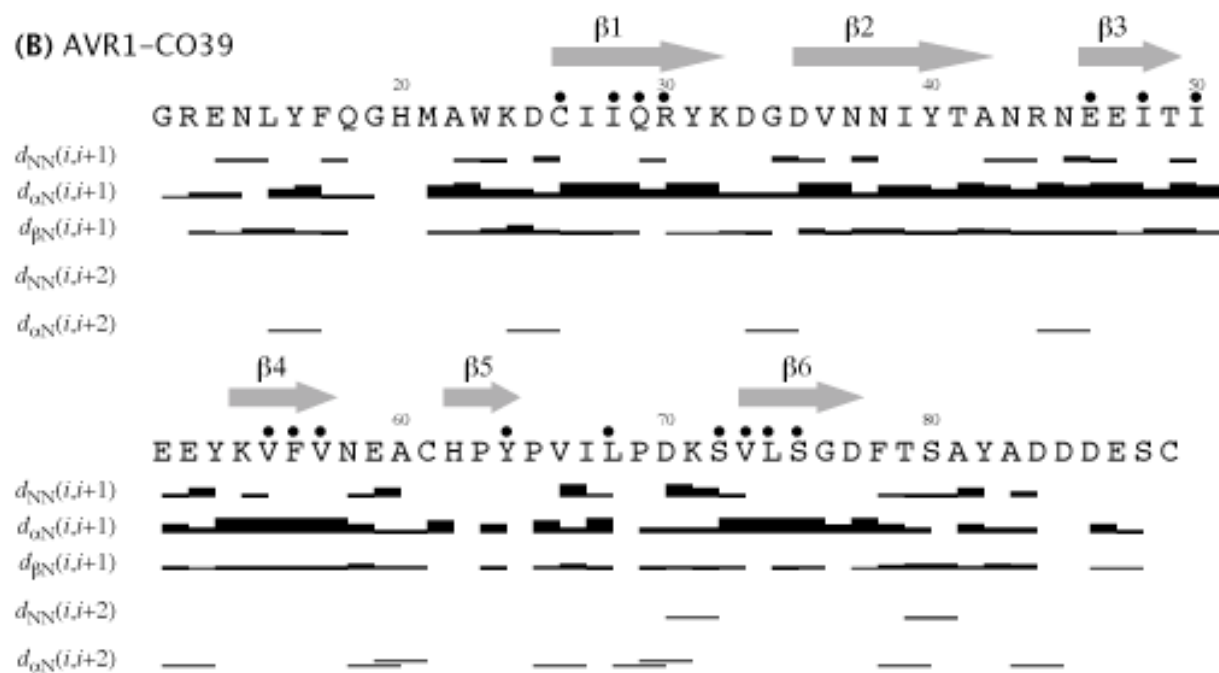

Supplement: S3 Fig — The line width is proportional to the NOE intensity. The dots (•) indicate slow exchange NH observed in 2D-NOESY in D2O. Grey arrows indicate the ß-strands determined from the structure analysis (vide infra). (PDF) [file ppat.1005228.s007.pdf]

**A**

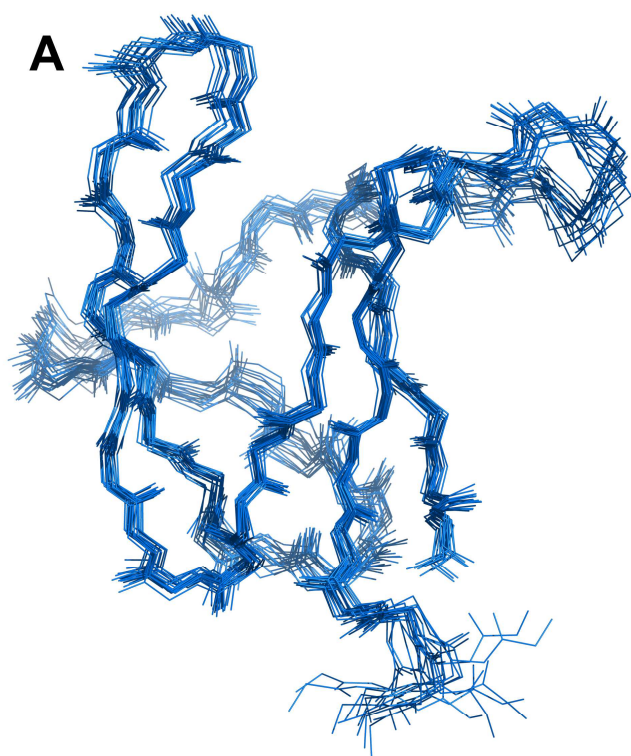

**B**

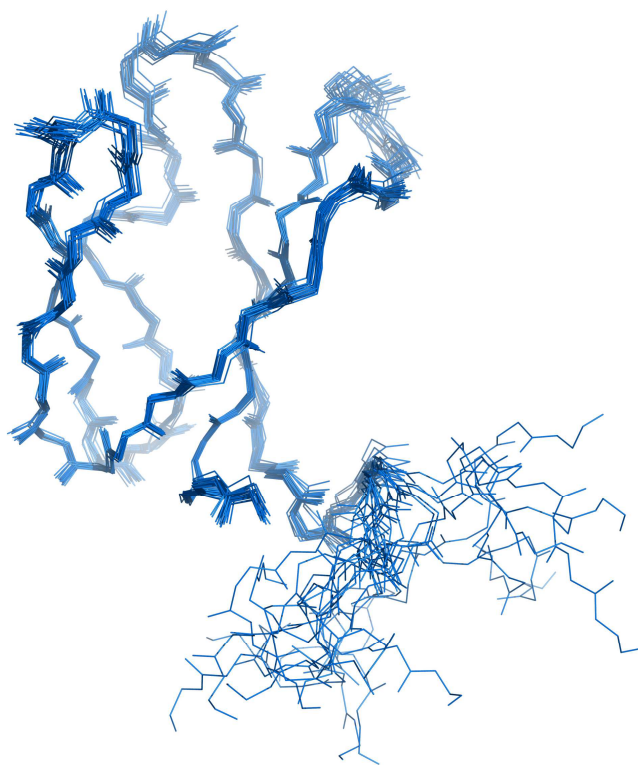

Supplement: S4 Fig — Superposition of the backbone atoms of the 20 lowest energy conformers used to calculate the final structures. Only mature chains are shown, from residues Ala20 and Trp23 for AVR-Pia and AVR1-CO39, respectively. (PDF) [file ppat.1005228.s008.pdf]

[illegible]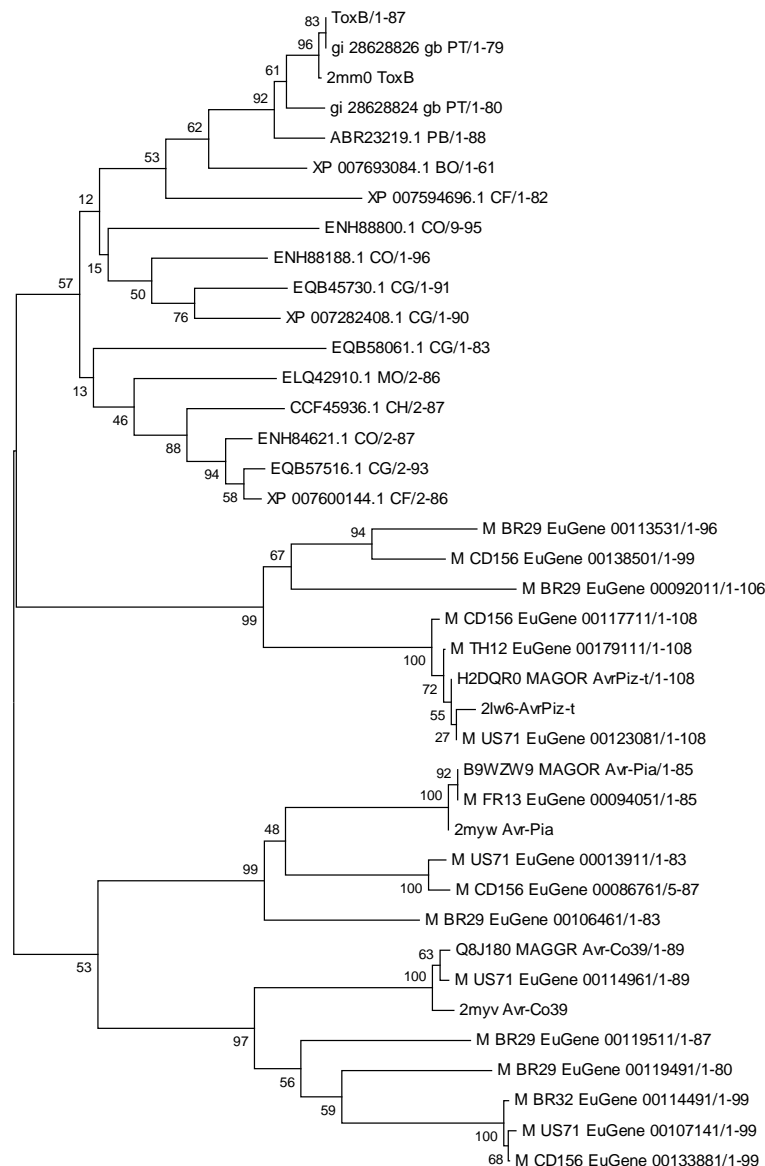

Supplement: S5 Fig — A) Homologs of AVR1-CO39, AvrPiz-t and AVR-Pia identified by Blast in M. oryzae and M. grisea genomes and ToxB homologs identified by Psi-Blast in the GeneBank database were aligned to the structural alignment of mature ToxB, AVR1-CO39, AvrPiz-t and AVR-Pia. (B) A diversity tree was constructed by the neighbor-joining method using the alignment in (A). It highlights the high diversity of MAX-effector homologs. Branch supports are based on 1000 bootstraps and horizontal branch length reflects sequence divergence. Accession numbers of non-Magnaporthe sequences were completed by a 2 letter identifier for the species: BO for Bipolaris oryzae, CF is for Colletotrichum fioriniae, CH for C. higgensianum, CG for C. gloeosporioides, CO for C. orbiculare, LM for Lepthosphaeria maculans, PT for Pyrenophora tritici-repentis and PB for Pyrenophora bromi. (PDF) [file ppat.1005228.s009.pdf]

A

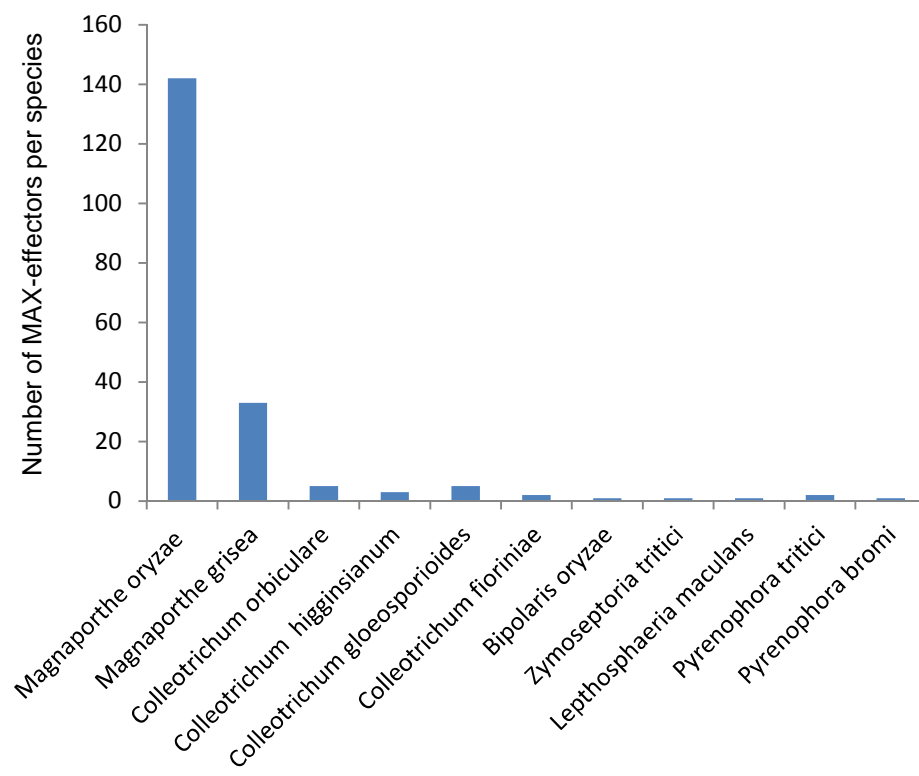

B

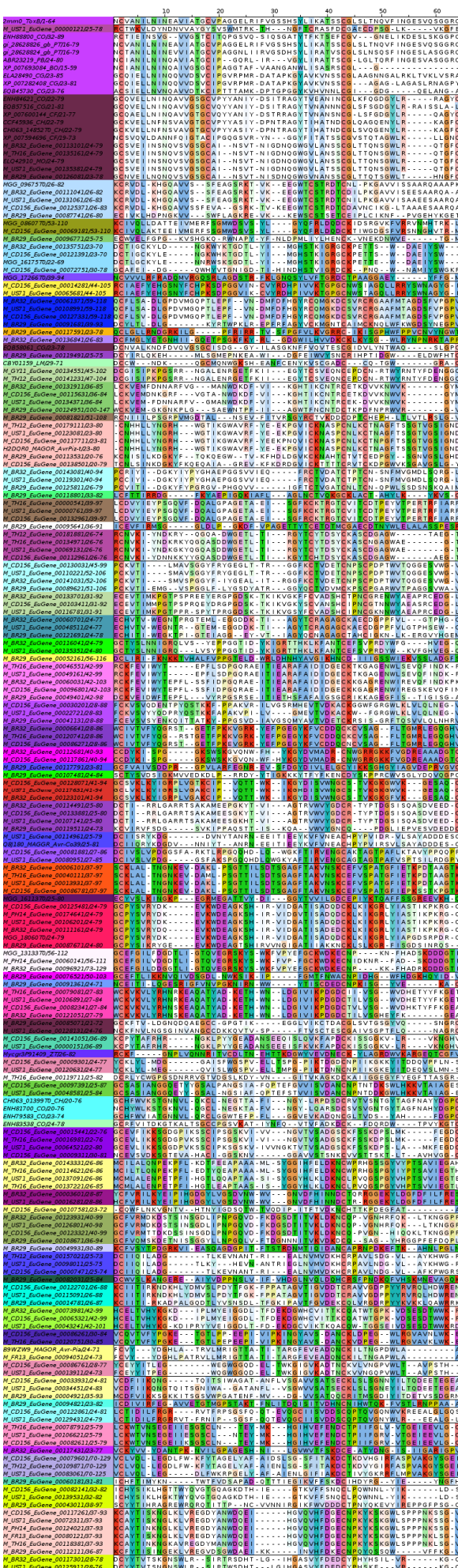

C

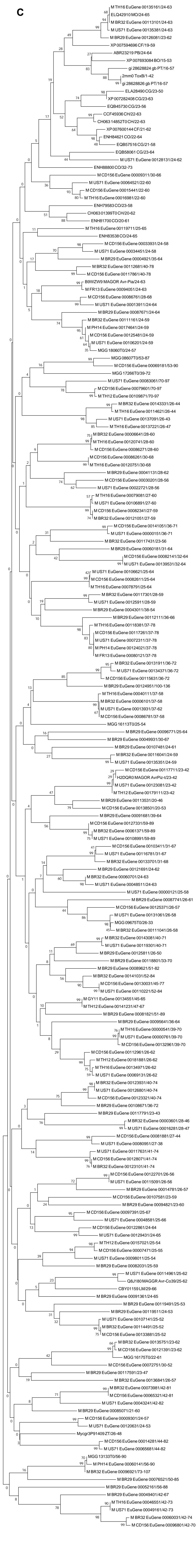

Supplement: S6 Fig — (A) Histogram showing the numbers of MAX-effectors identified by an HMM pattern search in a non-redundant database comprising the small secreted proteins of 25 ascomycete fungi and of 8 additional M. oryzae and one M. gisea isolate. (B) MAX-effectors were aligned to the structural alignment of mature ToxB, AVR1-CO39, AvrPiz-t and AVR-Pia and gaps were removed. (C) A diversity tree was constructed by the neighbor-joining method using the alignment in (B). Branch supports are based on 1000 bootstraps and horizontal branch length reflects sequence divergence. Accession numbers of non-Magnaporthe sequences were completed by a 2 letter identifier for the species: BO for Bipolaris oryzae, CF for Colletotrichum fioriniae, CH for C. higgensianum, CG for C. gloeosporioides, CO for C. orbiculare, LM for Lepthosphaeria maculans, PT for Pyrenophora tritici-repentis PB for Pyrenophora bromi and ZT for Zymoseptoria tritici. (PDF) [file ppat.1005228.s010.pdf]

A

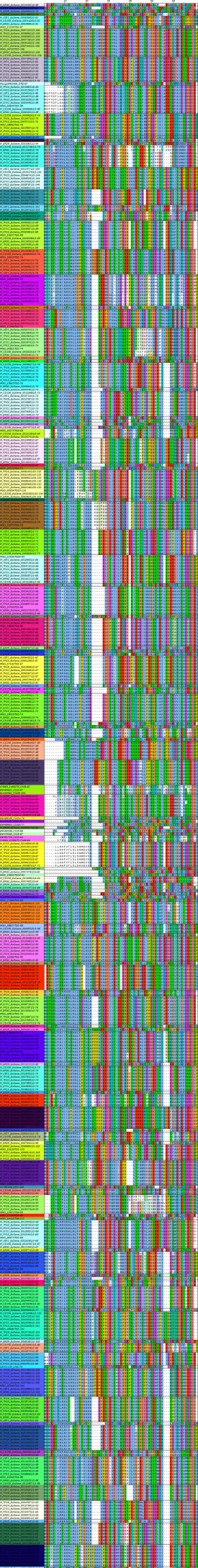

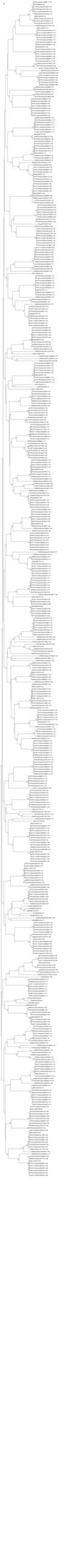

Supplement: S8 Fig — (A) MAX-effectors identified by an HMM pattern search in a redundant database comprising the small secreted proteins of 25 ascomycete fungi, 8 additional M. oryzae and one M. gisea isolate were aligned to the structural alignment of mature ToxB, AVR1-CO39, AvrPiz-t and AVR-Pia and gaps were removed. (B) A diversity tree was constructed by the neighbor-joining method using the alignment in (A). Branch supports are based on 1000 bootstraps and horizontal branch length reflects sequence divergence. Accession numbers of non-Magnaporthe sequences were completed by a 2 letter identifier for the species: BO for Bipolaris oryzae, CF for Colletotrichum fioriniae, CH for C. higgensianum, CG for C. gloeosporioides, CO for C. orbiculare, GF for Fusarium fujcuroi, LM for Lepthosphaeria maculans, PT for Pyrenophora tritici-repentis, PB for Pyrenophora bromi and ZT for Zymoseptoria tritici. (PDF) [file ppat.1005228.s012.pdf]

**A**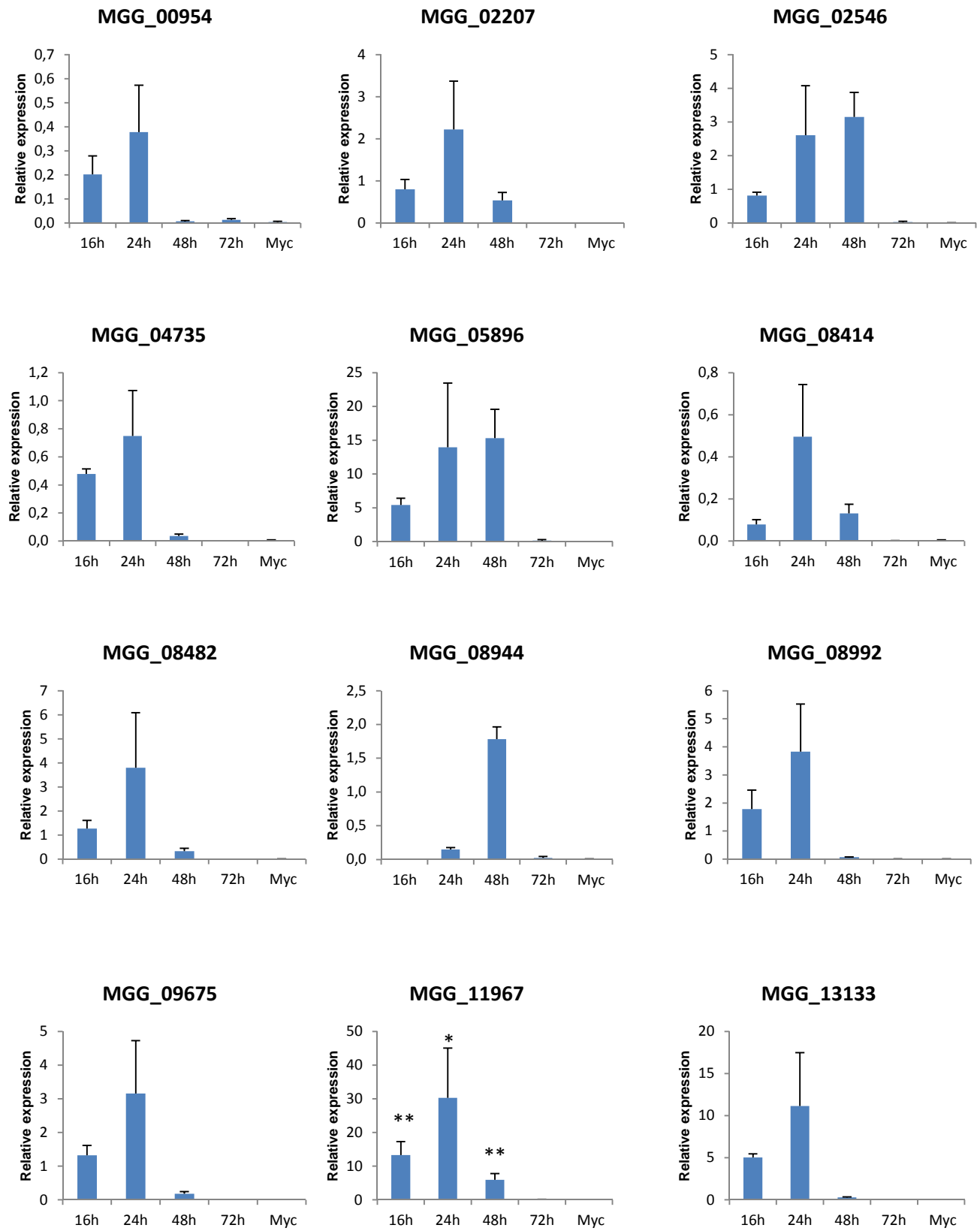

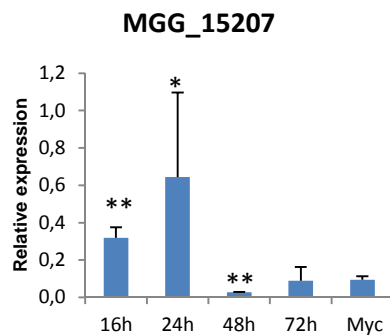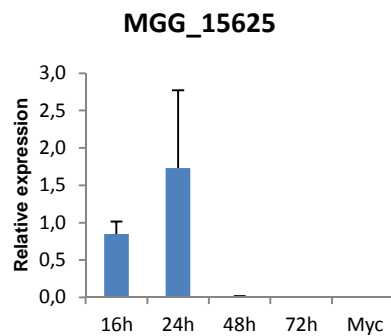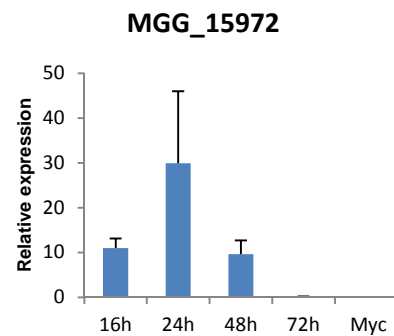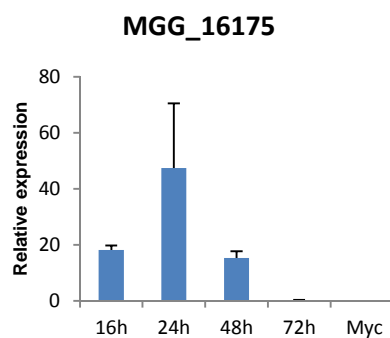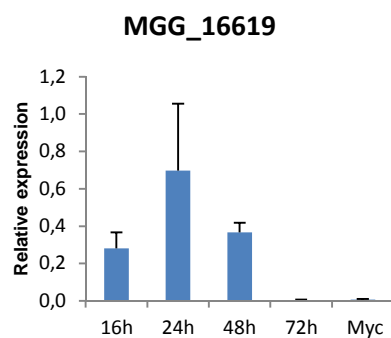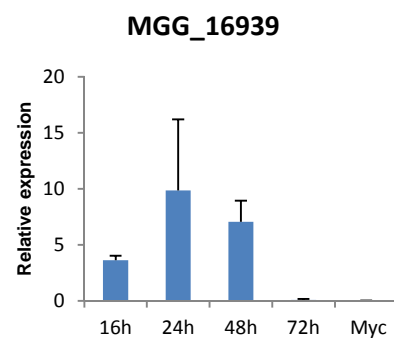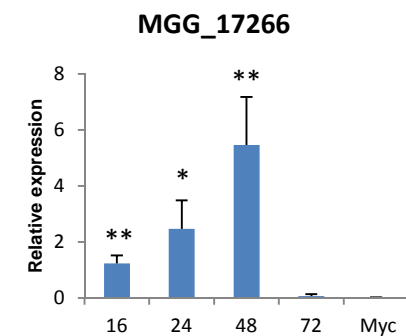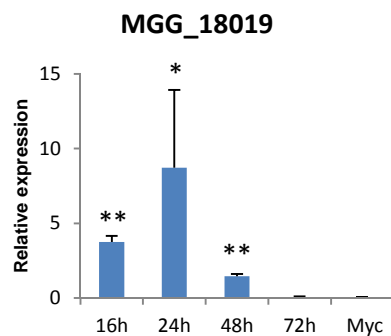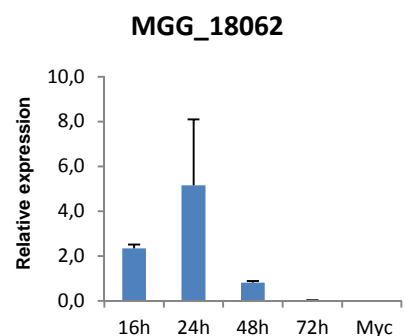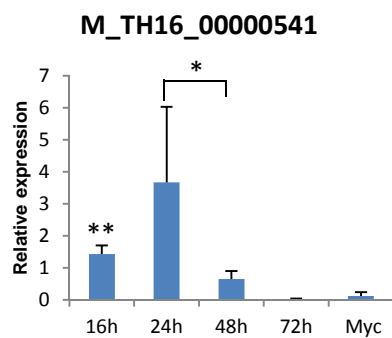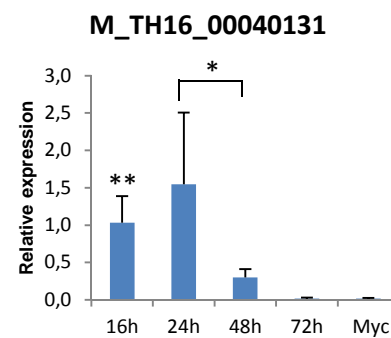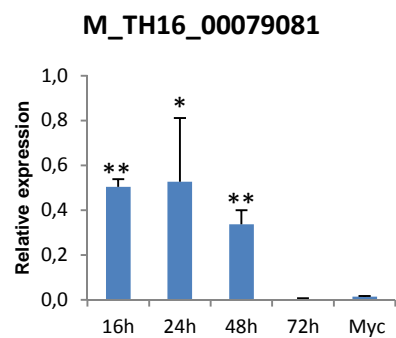

**M\_TH16\_00079311**

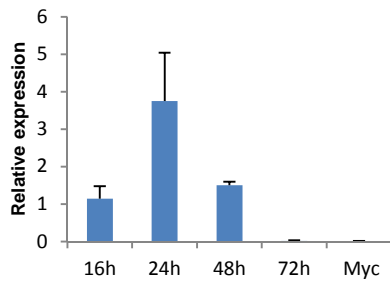

**M\_TH16\_00104561**

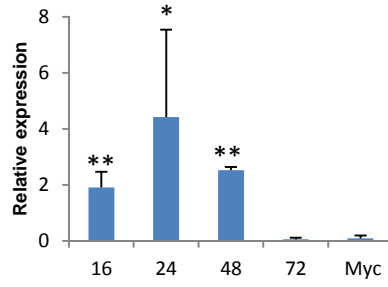

**M\_TH16\_00119711**

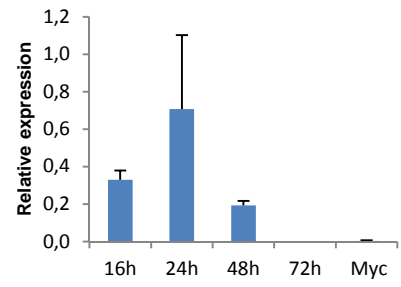

**M\_TH16\_00120731**

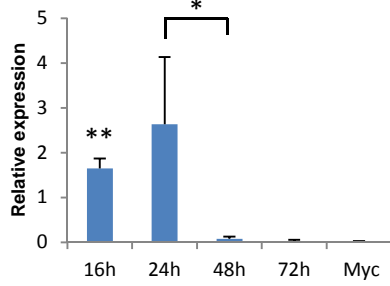

**M\_TH16\_00124981**

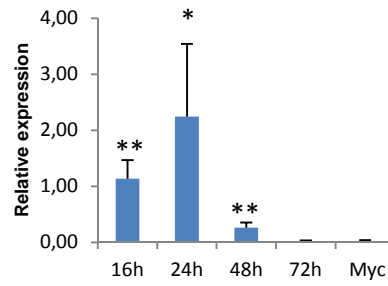

**M\_TH16\_00127871**

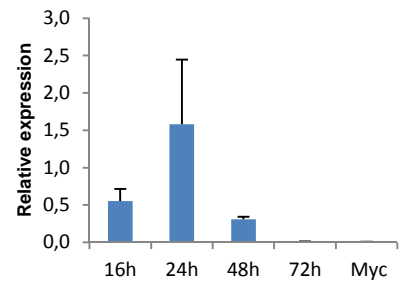

**B**

**M\_TH16\_00027411**

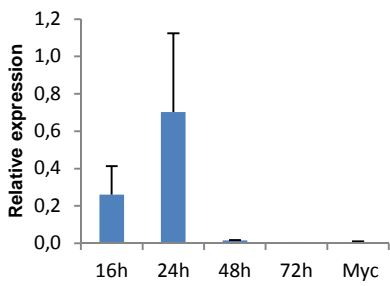

**M\_TH16\_00034081**

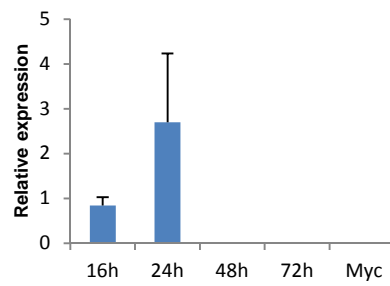

**M\_TH16\_00136331**

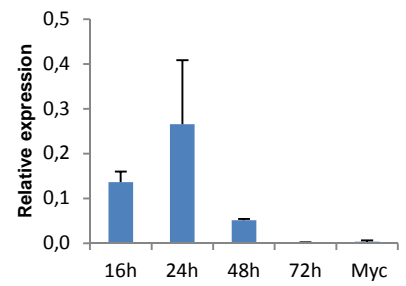

**C**

**MGG\_14793**

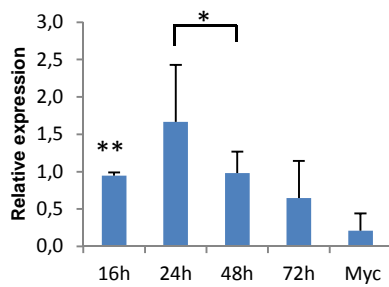

**MGG\_07184**

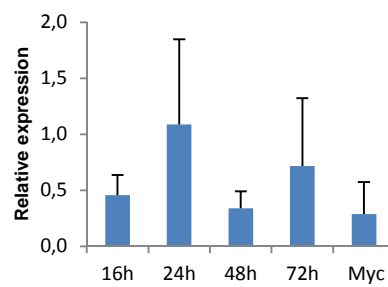

**D**

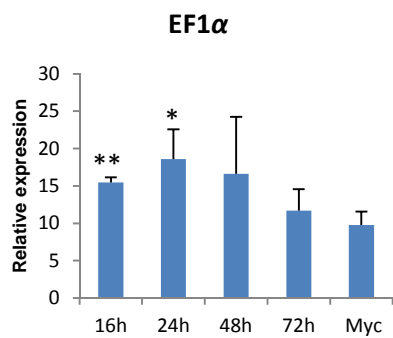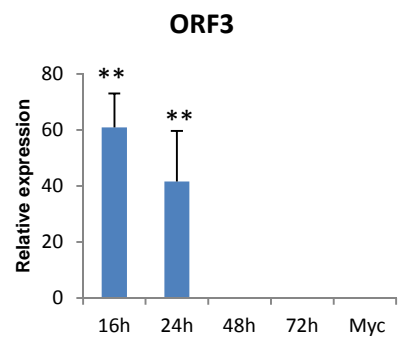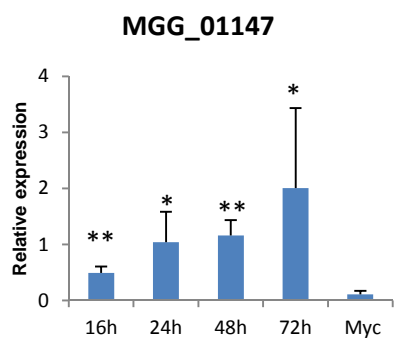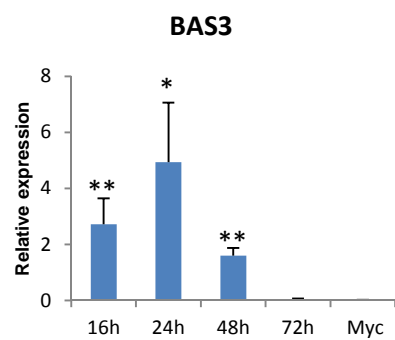

Supplement: S9 Fig — mRNA levels of M. oryzae genes coding for MAX-effectors (A, B and C) and marker genes (D) was determined by q-RT-PCR in rice leaf samples harvested 16, 24, 48 or 72 h after inoculation and mycelium grown liquid medium for 72 hours. (A) Infection specific MAX-effectors identified in the HMM search, (B) infection specific MAX-effectors identified in the Psi Blast search but nor in the HMM search, (C) constitutively expressed MAX-effectors identified in the HMM search and (D) marker genes for appressorium and very early infection (ORF3 of the ACE1 cluster, MGG_08381), biotrophic infection (BAS3, MGG_11610), late infection (MGG_01147), constitutive expression (EF1α, MGG_03641). Relative expression levels were calculated by using expression of a constitutively expressed Actin (MGG_03982) as a reference. Mean values and standard deviation were calculated from three independent biological samples.The analyzed genes, were in most cases not or extremely weakly expressed in the mycelium. For genes with significant expression in the mycelium (ratio gene versus actine > 0,01) a T-test was performed to determine if in planta expression was significantly different from expression in the mycelium. In these cases (MGG_11967, MGG_14793, MGG_15207, MGG_17266, MGG_18019, M_TH16_00000541, M_TH16_00040131, M_TH16_00079081, M_TH16_00104561, M_TH16_00120731, M_TH16_00124981), a star or two stars (* or **) mark conditions where the expression was different from expression in the mycelium at respectively p<0,05 or p<0,005. (PDF) [file ppat.1005228.s013.pdf]

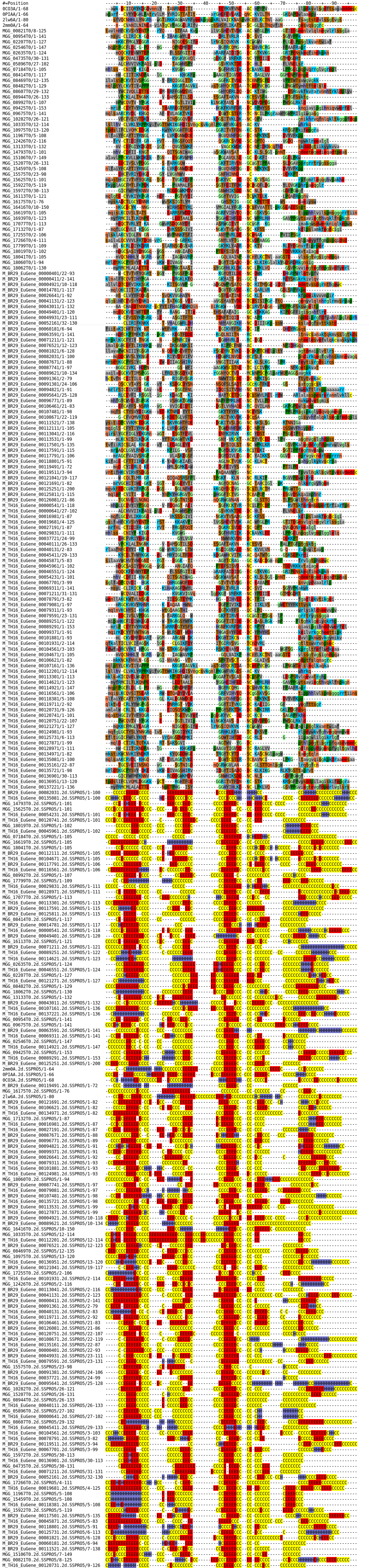

Supplement: S10 Fig — The secondary structures of the MAX-effectors from the 70–15, TH16 and BR29 genomes was predicted with SSPRO5 [93].The predictions are shown at the bottom of the figure and are aligned onto the corresponding primary sequence alignment shown at the top of the figure. Sequence identifiers for the secondary structure predictions are suffixed with ".2d.SSPRO5". Blue"H", red "E" and yellow "C" correspond respectively to helix, extended sheet and coil predictions. The sequences of the 4 MAX effectors with experimentally determined structures are displayed at the top of the multiple sequence alignment and, for clarity, the alignment positions corresponding to shared gaps in the known structures were removed. (TIF) [file ppat.1005228.s014.tif]
