# Supplementary material for: Structure Analysis Uncovers a Highly Diverse but Structurally Conserved Effector Family in Phytopathogenic Fungi
Source: PLoS Pathog. 2015 Oct 27;11(10):e1005228. doi: 10.1371/journal.ppat.1005228 (PMC4624222; doi:10.1371/journal.ppat.1005228)
Supplement: S7 Fig — (A) Histogram showing the numbers of MAX-effectors identified by an HMM pattern search in a non-redundant UniRef90 database. (B) MAX-effectors identified by HMM pattern search were aligned to the structural alignment of mature ToxB, AVR1-CO39, AvrPiz-t and AVR-Pia. (C) A diversity tree was constructed by the neighbor-joining method using the alignment in (B). This highlights the high diversity of MAX-effector homologs. Branch supports are based on 1000 bootstraps and horizontal branch length reflects sequence divergence. Accession numbers contain the following information on the species: MAGGR, MAGO7, MAGOP and MAGOR are from M. oryzae, COLGC and COLGN from C. gloeosporioides, COLHI from Colletotrichum higginsianum, 9PEZI from C. fioriniae and COLOR from Colletotrichum orbiculare, 9PLEO fromP. tritici-repentis or P. bromi, ARTOA from Arthrobotrys oligospora, COCMI from Bipolaris oryzae, LEPMJ from Leptosphaeria maculans, MYCGM from Zymoseptoria tritici, 9PSED from Pseudomonas sp. StFLB209 and SOLLC from Solanum lycopersicum. (PDF) [file ppat.1005228.s011.pdf]

A

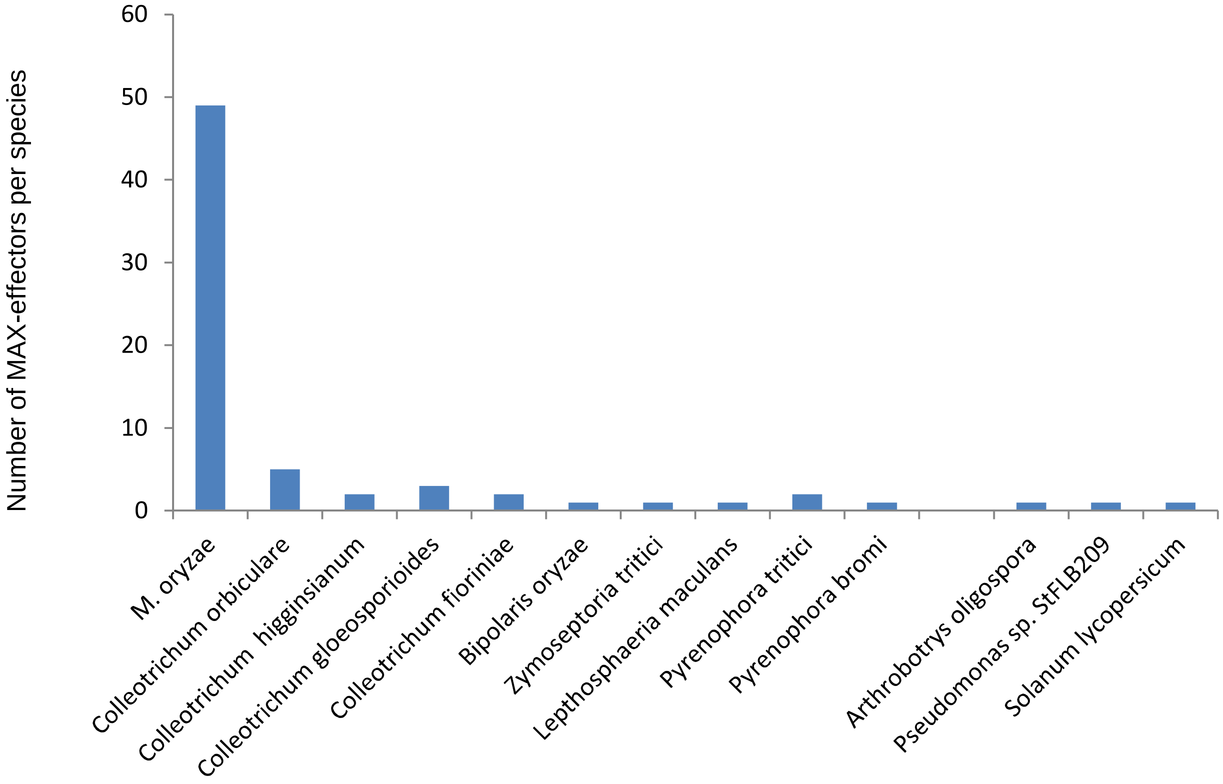

Number of MAX-effectors per species
60
50
40
30
20
10
0
M. oryzae
Colleotrichum orbiculare
Colleotrichum higginsianum
Colleotrichum gloeosporioides
Colleotrichum fioriniae
Bipolaris oryzae
Zymoseptoria tritici
Lepthosphaeria maculans
Pyrenophora tritici
Pyrenophora bromi
Arthrobotrys oligospora
Pseudomonas sp. StFLB209
Solanum lycopersicum

B

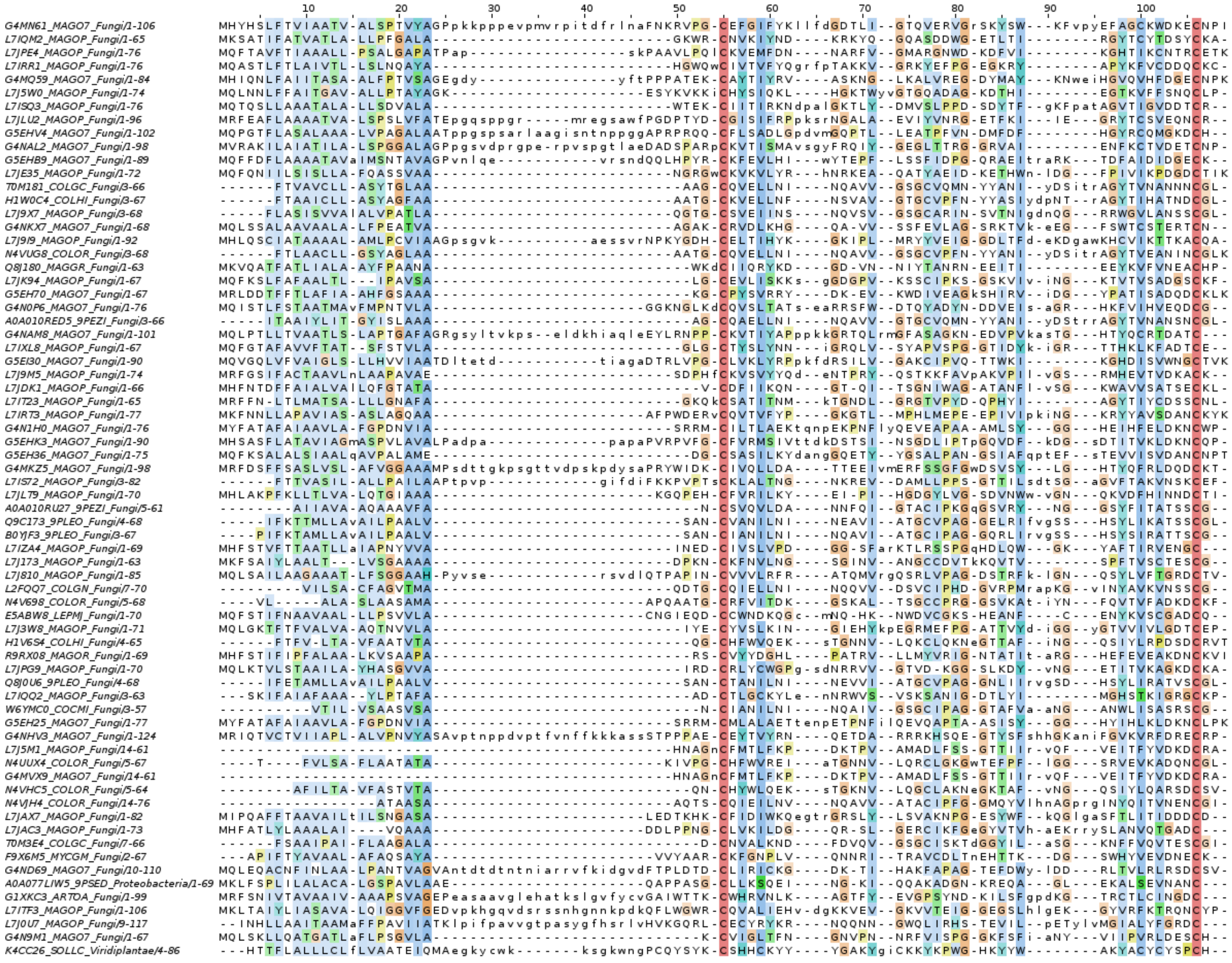

C

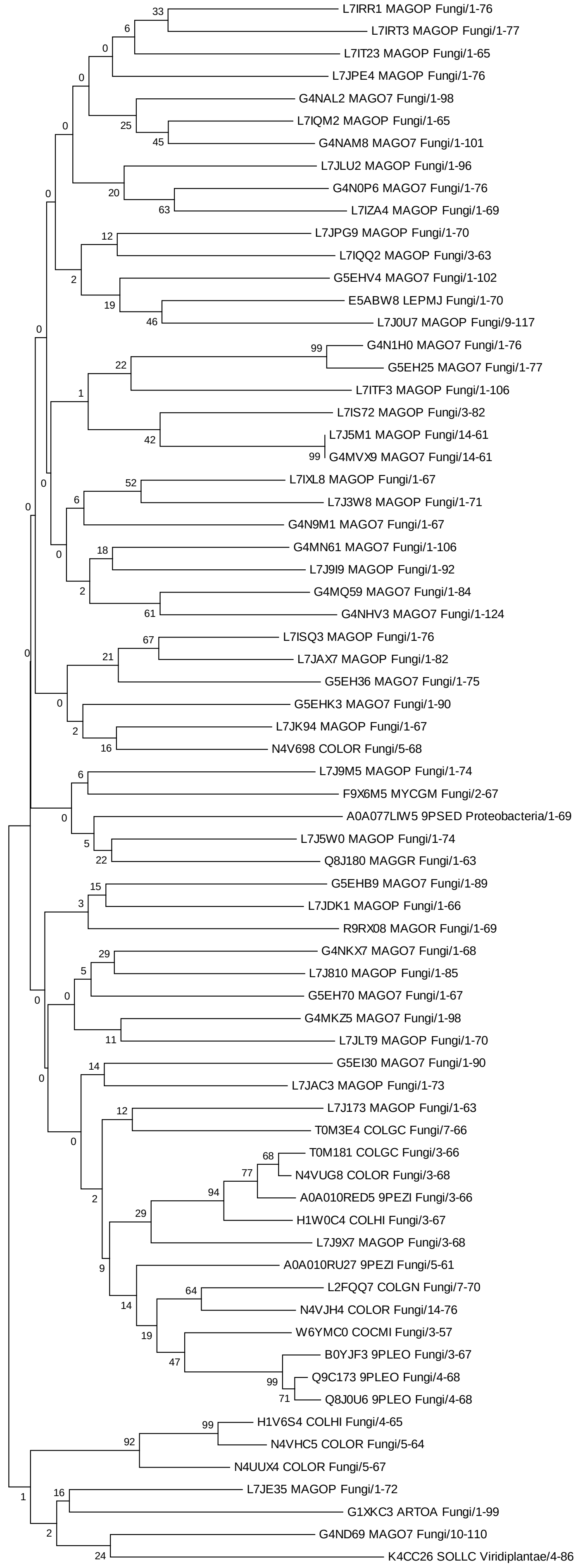
L7IRR1 MAGOP Fungi/1-76
L7IRT3 MAGOP Fungi/1-77
L7IT23 MAGOP Fungi/1-65
L7JPE4 MAGOP Fungi/1-76
G4NAL2 MAGO7 Fungi/1-98
L7IQM2 MAGOP Fungi/1-65
G4NAM8 MAGO7 Fungi/1-101
L7JLU2 MAGOP Fungi/1-96
G4N0P6 MAGO7 Fungi/1-76
L7IZA4 MAGOP Fungi/1-69
L7JPG9 MAGOP Fungi/1-70
L7IQQ2 MAGOP Fungi/3-63
G5EHV4 MAGO7 Fungi/1-102
E5ABW8 LEPMJ Fungi/1-70
L7J0U7 MAGOP Fungi/9-117
G4N1H0 MAGO7 Fungi/1-76
G5EH25 MAGO7 Fungi/1-77
L7ITF3 MAGOP Fungi/1-106
L7IS72 MAGOP Fungi/3-82
L7J5M1 MAGOP Fungi/14-61
G4MVX9 MAGO7 Fungi/14-61
L7IXL8 MAGOP Fungi/1-67
L7J3W8 MAGOP Fungi/1-71
G4N9M1 MAGO7 Fungi/1-67
G4MN61 MAGO7 Fungi/1-106
L7J9I9 MAGOP Fungi/1-92
G4MQ59 MAGO7 Fungi/1-84
G4NHV3 MAGO7 Fungi/1-124
L7ISQ3 MAGOP Fungi/1-76
L7JAX7 MAGOP Fungi/1-82
G5EH36 MAGO7 Fungi/1-75
G5EHK3 MAGO7 Fungi/1-90
L7JK94 MAGOP Fungi/1-67
N4V698 COLOR Fungi/5-68
L7J9M5 MAGOP Fungi/1-74
F9X6M5 MYCGM Fungi/2-67
A0A077LIW5 9PSED Proteobacteria/1-69
L7J5W0 MAGOP Fungi/1-74
Q8J180 MAGGR Fungi/1-63
G5EHB9 MAGO7 Fungi/1-89
L7JDK1 MAGOP Fungi/1-66
R9RX08 MAGOR Fungi/1-69
G4NKX7 MAGO7 Fungi/1-68
L7J810 MAGOP Fungi/1-85
G5EH70 MAGO7 Fungi/1-67
G4MKZ5 MAGO7 Fungi/1-98
L7JLT9 MAGOP Fungi/1-70
G5EI30 MAGO7 Fungi/1-90
L7JAC3 MAGOP Fungi/1-73
L7J173 MAGOP Fungi/1-63
T0M3E4 COLGC Fungi/7-66
T0M181 COLGC Fungi/3-66
N4VUG8 COLOR Fungi/3-68
A0A010RED5 9PEZI Fungi/3-66
H1W0C4 COLHI Fungi/3-67
L7J9X7 MAGOP Fungi/3-68
A0A010RU27 9PEZI Fungi/5-61
L2FQQ7 COLGN Fungi/7-70
N4VJH4 COLOR Fungi/14-76
W6YMC0 COCMI Fungi/3-57
B0YJF3 9PLEO Fungi/3-67
Q9C173 9PLEO Fungi/4-68
Q8J0U6 9PLEO Fungi/4-68
H1V6S4 COLHI Fungi/4-65
N4VHC5 COLOR Fungi/5-64
N4UUX4 COLOR Fungi/5-67
L7JE35 MAGOP Fungi/1-72
G1XKC3 ARTOA Fungi/1-99
G4ND69 MAGO7 Fungi/10-110
K4CC26 SOLLC Viridiplantae/4-86
